# A comprehensive evaluation of large language models in mining gene relations and pathway knowledge

**DOI:** 10.1002/qub2.57

**Published:** 2024-06-21

**Authors:** Muhammad Azam, Yibo Chen, Micheal Olaolu Arowolo, Haowang Liu, Mihail Popescu, Dong Xu

**Affiliations:** ^1^ Department of Electrical Engineering and Computer Science University of Missouri Columbia Missouri USA; ^2^ Bond Life Sciences Center University of Missouri Columbia Missouri USA; ^3^ Institute for Data Science and Informatics University of Missouri Columbia Missouri USA; ^4^ Department of Biomedical Informatics Biostatistics and Medical Epidemiology University of Missouri Columbia Missouri USA

**Keywords:** biomedical text mining, gene–gene interaction, KEGG pathway, large language model

## Abstract

Understanding complex biological pathways, including gene–gene interactions and gene regulatory networks, is critical for exploring disease mechanisms and drug development. Manual literature curation of biological pathways cannot keep up with the exponential growth of new discoveries in the literature. Large‐scale language models (LLMs) trained on extensive text corpora contain rich biological information, and they can be mined as a biological knowledge graph. This study assesses 21 LLMs, including both application programming interface (API)‐based models and open‐source models in their capacities of retrieving biological knowledge. The evaluation focuses on predicting gene regulatory relations (activation, inhibition, and phosphorylation) and the Kyoto Encyclopedia of Genes and Genomes (KEGG) pathway components. Results indicated a significant disparity in model performance. API‐based models GPT‐4 and Claude‐Pro showed superior performance, with an F1 score of 0.4448 and 0.4386 for the gene regulatory relation prediction, and a Jaccard similarity index of 0.2778 and 0.2657 for the KEGG pathway prediction, respectively. Open‐source models lagged behind their API‐based counterparts, whereas Falcon‐180b and llama2‐7b had the highest F1 scores of 0.2787 and 0.1923 in gene regulatory relations, respectively. The KEGG pathway recognition had a Jaccard similarity index of 0.2237 for Falcon‐180b and 0.2207 for llama2‐7b. Our study suggests that LLMs are informative in gene network analysis and pathway mapping, but their effectiveness varies, necessitating careful model selection. This work also provides a case study and insight into using LLMs das knowledge graphs. Our code is publicly available at the website of GitHub (Muh‐aza).

## 1 INTRODUCTION

Biological pathways, encompassing gene–gene interactions, metabolic networks, and gene regulatory networks, are complex systems integral to processes such as molecular signaling [1]. Their understanding is crucial in deciphering disease mechanisms and advancing drug development. Biological pathway information is contained in the literature. Manual curations of the literature produced databases like the Kyoto Encyclopedia of Genes and Genomes (KEGG) [2], which are instrumental in systematically organizing and visualizing these networks. However, extracting knowledge from the biomedical text is labor‐intensive and time‐consuming, which has led to the rise of automated mining techniques that distill valuable insights from the extensive biomedical literature [3].

A major development in natural language processing is the emergence of large‐scale language models (LLMs), characterized by their enormous parameter sizes and training on extensive text corpora [4]. Their ability to generate coherent, contextually relevant text makes them particularly suitable for biomedical text generation and mining [5]. Many LLMs have been developed for biomedical studies, as shown in Table 1. Models like BioLinkBERT have shown the potential of LLMs in biomedical text mining [6]. Most LLMs adopt the *pre‐train*, *prompt*, *and predict paradigm*, which involves enhancing problem statements with specific instructions for learning in prompts [7]. Among the most recent LLMs, the GPT‐4 is recognized for its advanced abilities in language comprehension and generation, conversational AI, code generation, and language translation tasks. Its predecessor, GPT‐3.5, established the groundwork for these advanced tasks [8]. Claude and Claude Instant have shown proficiency in context‐aware responses, especially in extended interactions [9] while the Cohere Playground excels in text classification, sentiment analysis, and summarization [10]. In the open‐source LLMs, Codellama‐34 is known for its efficiency in instruction‐based tasks [11], and the WizardLM series excels in knowledge retrieval and processing [12]. Falcon‐180b specializes in conversational AI [13] and Mistral‐7b is tailored for instruction‐following tasks. The Vicuna series [14] and the llama2 series [15] are used in a variety of language modeling and text generation applications. Qwen‐14b is recognized for its efficiency in medium‐scale language tasks [16].

**TABLE 1:** Examples of LLMs and their key features.

Model name	Specialization	Notable applications	Technical detail
BioLinkBERT	Biomedical text mining	Extensive biomedical corpus training	Utilizes domain‐adaptive pretraining on biomedical data
GPT‐4	Advanced language tasks	Language comprehension, generation, code, translation	Zero‐shot and few‐shot learning capabilities, dynamic attention mechanism
GPT‐3.5	Foundational language tasks	Groundwork for advanced tasks	Introduction of in‐context learning for task adaptation
Claude	Context‐aware response	Extended interactions	Adaptive language models for real‐time context tracking
Claude instant	Context‐aware response	Speedy interactions	Optimizations for latency reduction in conversational models
Cohere	Text analysis	Classification, sentiment analysis, summarization	Focuses on efficient transfer learning for custom tasks
Codellama‐34	Instruction‐based tasks	Efficient instruction following	Designed for high efficiency in instruction‐following with minimal data
WizardLM series	Knowledge retrieval	Comprehends and follows complex instructions	An evolutionary approach to gradually increase the complexity of instructions
Falcon‐180b	Conversational AI	Advanced conversational abilities	High‐capacity model tailored for nuanced conversational understanding
Mistral‐7b	Instruction following	Tailored for following instructions	Efficiency in processing with emphasis on privacy‐preserving techniques
Vicuna series	Language modeling	Versatile text generation applications	Modular design for scalable deployment across various computing environments
llama2 series	Language modeling	Diverse language modeling and generation	Open‐source accessibility, emphasizes community‐driven enhancements
Qwen‐14b	Language modeling	Efficiency in medium‐scale language and coding tasks	Specializes in computational efficiency and low‐resource training

Abbreviation: LLMs, large‐scale language models.

Although LLMs are often used as knowledge bases or knowledge graphs to retrieve information, their accuracies are rarely studied, even when ground‐truth data are available. This can create a false sense of confidence and a lack of clarity, hindering applications and improvement of LLMs. To address this issue, we aim to systematically evaluate the capacities of LLM in extracting crucial biomedical information, such as pathway knowledge, gene interactions, and regulatory relationships, in a case study. We comprehensively assessed API‐based models and open‐source models based on their capacities in predicting gene regulatory relationships and KEGG pathway components.

The remainder of this paper is structured as follows: Section 2 presents the results of our experiments. Section 3 summarizes key findings and outlines future research directions. Finally, Section 4 provides method details.

## 2 RESULTS

**FIGURE 1:**
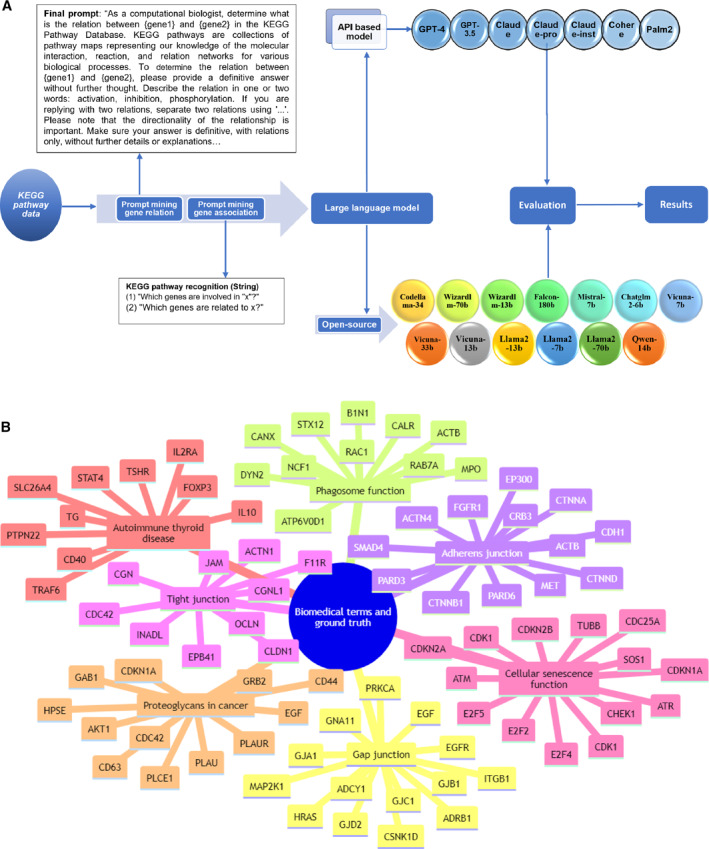
An assessment workflow for LLMs in analyzing biomedical data. (A) The process starts with prompts to LLMs for gene relationship prediction and recognition of genes associated with biomedical terms. The LLMs used in this study include both API‐based and open‐source models. The results were evaluated comprehensively and rigorously. (B) Ground‐truth genes associated with the seven biomedical terms used in this study. API, application programming interface; LLMs, large‐scale language models.

Figure 1 shows a pipeline used to evaluate 21 LLMs. We conducted a two‐part analysis, the first focusing on gene regulatory relations (specifically activation, inhibition, and phosphorylation) and the second on KEGG pathway components (predicting gene names in the form of strings in a pathway). We critically evaluated 21 LLMs against a dataset from the KEGG pathway. Our approach involved using specially crafted prompts to extract data from each LLM. We first systematically assessed each model’s effectiveness in identifying and interpreting gene regulatory relations using key statistical indicators, such as precision, recall, and F1 scores. We then conducted a similar assessment for retrieving genes involved in a given KEGG pathway. The insights garnered from this study offer a detailed perspective on the capabilities and limitations of LLMs in retrieving biological knowledge.

### 2.1 Evaluations of gene regulatory relations

We assessed the effectiveness of the 21 LLMs in identifying gene regulatory relationships, encompassing activation, inhibition, and phosphorylation, against a dataset from the KEGG pathway, which included 200 gene pairs and their known relationships (Table S1). We tested various prompts. Table S2 shows seven example prompts we explored and the final one we selected based on preliminary performance tests. As an example, Table S3 shows 15 gene pairs, including relationships of activation, inhibition, and phosphorylation, with five pairs for each category. Varying gene regulatory relationships from different prompts were obtained for these 15 gene pairs with the default LLM temperature of 0.9 (Table S4). The temperature in an LLM is a parameter used to control the randomness of the generated output. A higher temperature leads to more randomness, while a lower temperature leads to more deterministic output. To explore the effects of temperature, we applied different temperatures using the final selected prompt on the 15 gene pairs, as shown in Table S5. It shows that a temperature of 0.9 provides the most informative and valuable results.

Among the 15 gene pairs, most LLMs correctly predicted the activation relation from CCR5 to GNB3 under various prompts. A simple Google Search using “CCR5 GNB3 activation” on 18 February 2024, yielded 5760 results. In contrast, few LLMs predicted the phosphorylation relation from GRK3 to CXCR6. The same search using “GRK3 CXCR6 Phosphorylation” only obtained 1090 results. It appears that the chance to predict correctly may be related to the availability of related text.

**FIGURE 2:**
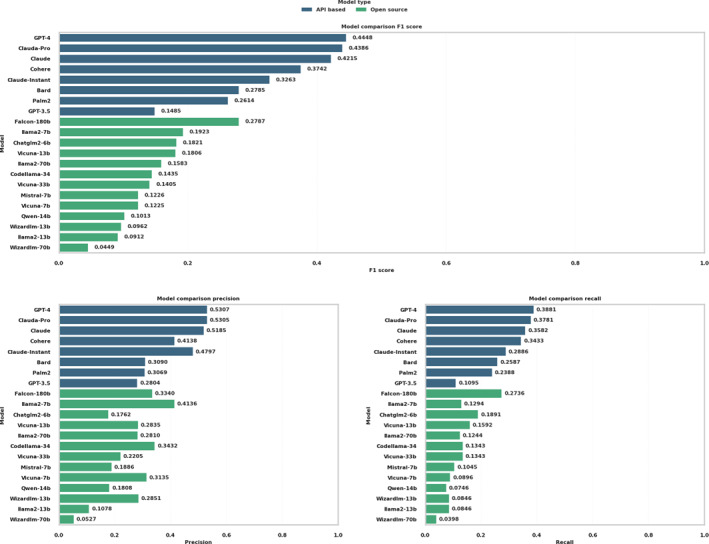
Comparative analysis of gene prediction accuracy across API‐based and open‐source models, measured by precision, recall, and F1 score. API, application programming interface.

Figure 2 shows the overall performance, with the final selected prompt and temperature of 0.9. A distinct performance gradient was observed between proprietary API‐based models and open‐source alternatives. Among the API‐based LLMs, GPT‐4 emerged as the best performer, exhibiting the highest F1 score of 0.4448 alongside recall and precision rates of 0.3881 and 0.5307, respectively. A close second, Claude‐Pro, registered a recall and precision of 0.3781 and 0.5305, respectively. Claude‐Pro also had an F1 score of 0.4386, underscoring its efficacy in the domain. Conversely, GPT‐3.5 displayed considerable limitations in this specific analytical setting, with a recall of 0.1095 and an F1 score of 0.1485, one of the poorest performers among the 21 LLMs. The remainder of the API‐based models, including Cohere, Claude, Palm2, Bard, and Claude‐Instant, demonstrated moderate capabilities, with their performance metrics clustering below the 0.4 threshold across all four measured indices.

On the open‐source front, the results were more varied, with none of the models surpassing the API‐based frontrunners. llama2‐7b highlighted the highest precision among its open‐source peers at 0.4136 (i.e., with the least hallucination), although it yielded modest recall and F1 scores at 0.1923 and 0.129, respectively. This discrepancy points to a considerable number of missed true positives. The rest of the open‐source models, including Codellama‐34, Mistral‐7b, Vicuna‐33b, Vicuna‐13b, llama2‐70b, and Falcon‐180b, demonstrated lower levels of recall and precision, with corresponding F1 scores below the 0.30 mark. Models such as Wizardly‐70b, Wizardly‐13b, Vicuna‐7b, llama2‐7b, llama2‐13b, and Qwen‐14 occupied the lower tier of performance, with Qwen‐14b, at the bottom. API‐based models, particularly Claude‐Pro and GPT‐4, demonstrated significantly better performance in detecting gene regulatory relationships than the open‐source models.

### 2.2 Evaluations of KEGG pathway recognition

In the second part of our study, we focused on the recognition of seven key biomedical terms within the KEGG Pathway: adherens junction, tight junction, GAP junction, cellular senescence, phagosome, proteoglycans in cancer, and autoimmune thyroid disease. We employed the 21 LLMs to assess their performance in identifying genes associated with these biomedical terms. We explored various prompts, as shown in Table S6, and the final one was based on *ad hoc* tests. We ran each query five times using the default settings of the 21 LLMs and assigned weights to the genes based on their occurrence. We used the Jaccard similarity index to compare the genes matched by the models with those in the established ground truth (Figure 1B).

#### 2.2.1 Adherens junction complex

**FIGURE 3:**
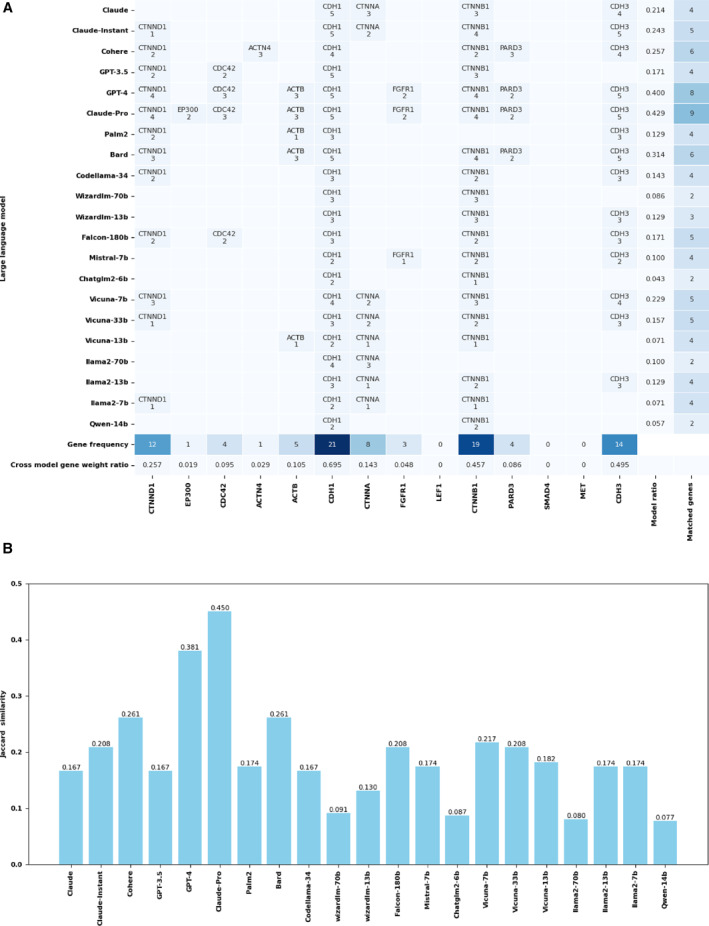
Gene predictions for adherens junctions. Part (A) displays the prediction accuracy and confidence scores from 21 API‐based and open‐source models. The bottom two rows show the frequency of correct gene occurrence among models (one or more correct predictions are counted as success for the model) and among all predictions (cross‐model gene weight ratio is the number of correct predictions divided by the number of all predictions, where each model has five predictions) for a given gene. The two columns on the far right show the frequency of correct gene occurrence in all predictions (the number of correct predictions divided by the number of all predictions for the model, where each model has five predictions), and the number of correctly predicted genes (for a given model, one or more correct predictions from the model on the gene are counted as a success for that gene). Part (B) compares the models through Jaccard similarity scores, assessing their accuracy in matching gene sets specific to the adherens junction. API, application programming interface.

Figure 3 shows the assessment of the adherens junction complex, a pivotal factor in cellular adhesion and signal transduction. Figure 3A presents a comparison of the 21 LLMs. The figure maps the prediction accuracies of each model in identifying true genes associated with the adherens junction, including CDH1, CTNNB1, CTNNA, CTNND1, CDC42, CDH3, ACTN4, ACTB, EP300, FGFR1, LEF1, PARD3, MET, LEF1, and SMAD4. It also quantifies the likelihood for each gene to be predicted correctly within the respective models, which varies considerably. CDH1 was predicted correctly by all models, while some genes (MET, SMAD4, and LEF1) could not be predicted at all by any model.

As LLMs typically used the Common Crawl dataset [17], any web materials open to the public, such as PubMed abstracts [18] and publications at the PMC Open Access [19], may be included in the training text of LLMs. The result in Figure 3 may reflect the availability of relevant text. For example, after searching “CDH1 Adherens” in PubMed on 15 January 2024, 150 hits were obtained. In contrast, our search for “MET Adherens”, “SMAD4 Adherens”, and “LEF1 Adherens” yielded only 50, 10, and 26 hits, respectively. MET had more hits probably because it is not a unique name, as it also represents the amino acid methionine.

The Jaccard similarity index in Figure 3B offers a comprehensive analysis of the model performance. This index measures the similarity between the predicted genes of each LLM and a reference set of known genes associated with the adherens junction, thereby providing a statistical basis for comparing their accuracies. All LLMs generated some false positives, as shown in Table 2, where we examined the unmatched genes to the known genes in the adherens junction pathway from each model. With these results, we calculated the precision, recall, and F1 score.

**TABLE 2:** Twenty‐one LLM false positive predictions and their performance in the adherens junction.

Model	False positive genes	# of unmatched genes	Match gene	Precision	Recall	F1
Claude	CTDNND1, CDC, ACTN1, ACT, PVRL1, MLLT4, LIMA1, PRKCA, MLLT4, VCL, DSP	11	4	0.2857	0.2857	0.2857
Claude‐instant	CDC, ACTN, ACT, PLEKHA7, RHOA, LMO7, DSG1, RAC1, VCL, AFADIN	10	5	0.3333	0.3571	0.3448
Cohere	CTNNA1, CD42, ACB, EPHA2, FBLN1, LED1, MYC, PLXNA2, SND1	9	6	0.4000	0.4286	0.4138
GPT‐3.5	CTNNA1, JUP, Rac1, ZO‐2, JAM, ARVCF, ZO‐2, Zo1, RHOA, VCL, AFADIN	11	4	0.2857	0.2857	0.2857
GPT‐4	CAV2, PAK1, AMOT, RHOC, PAK2, CAV1, IQGAP1	7	8	0.5333	0.5714	0.5517
Claude‐Pro	TJP1, ACTG1, CREBBP, AFDN, SRC, VCL	6	9	0.6000	0.6429	0.6207
Palm2	CTNN, CDH20, CDH25, ACTG, CTNN, CDH30, CDH15, CDH25, RHO, PLXN, VC	11	4	0.3077	0.2857	0.2963
Bard	CTNNA1, ACTN1, F11R, CDH24, TJP1, ZO, JAM, PLXN, AF	9	6	0.4000	0.4286	0.4138
Codellama‐34	CTNNA1, CDH7, CTNN, CDH30, CDH15, CDH25, RHO, PLXN, CTNN, FBLN1, LED1	11	4	0.2857	0.2857	0.2857
Wizardlm‐70	JUP, Rac1, ZO‐2, JUP, ARVCF, ZO‐2, Zo1, RHOA, VCL, AFADIN, ARVCF, ZO‐2, Zo1	13	2	0.2000	0.1429	0.1667
Wizardlm‐13	CTNNA1, Rac1, ZO‐2, Zo1, ZO‐2, JAM, ARVCF, ZO‐2, Zo1, RHOA, VCL, AFADIN	12	3	0.2500	0.2143	0.2308
Falcon‐180b	CTNNA1, ITGB, MLLT4, F11R, CDH30, CDH15, CDH25, RHO, PLXN, VC	10	5	0.3333	0.3571	0.3448
Mistral‐7b	Rac1, ZO‐2, AMOT, ZO‐2, PLEKHA7, RHOA, LMO7, DSG1, RAC1, VCL	10	4	0.2857	0.2857	0.2857
Chatglm2‐6	JUP, Rac1, ZO‐2, JAM, ARVCF, ZO‐2, Zo1, RHOA, VCL, AFADIN, JUP, Rac1, ZO‐2	13	2	0.1818	0.1429	0.1600
Vicuna‐7b	Ra1, ZO‐2, CDH7, DH30, ZO‐2, RHOA, VCL, PLXN, CTNN, LXN	10	5	0.3571	0.3571	0.3571
Vicuna‐33b	CD42, ITGB, MLLT4, F11R, CDH30, CDH15, CDH25, RHO, PLXN, VC	10	5	0.3333	0.3571	0.3448
Vicuna‐13b	Rac1, ZO‐2, JM, ARVCF, ARVCF, ZO‐2, Zo1, RHOA, VCL, AFADIN, ARVCF	11	4	0.3333	0.2857	0.3077
llama2‐70b	CTNN1, CTND1, ZO‐2, CD3, AMOT, ATB, RHOA, VCL, AFADIN, ARVCF, ZO‐2, Zo1, RHOA	13	2	0.1538	0.1429	0.1481
llama2‐13b	RVC, ITGB, F1R, CDH30, CDH15, CDH25, RHO, PLXN, VC, ITGB	10	4	0.3077	0.2857	0.2963
llama2‐7b	ZO‐2, CD3, F11R, CDH30, CDH15, CDH25, RHO, PLXN, VC, PLXN, VC	11	4	0.3077	0.2857	0.2963
Qwen‐14b	ZO‐2, ITGB, MLLT4, RHOA, VCL, AFADIN, JUP, Rac1, ZO‐2, CDH30, CDH15, CDH25, RHO	13	2	0.1429	0.1429	0.1429

Abbreviation: LLM, large‐scale language model.

Overall, the API‐based models (the first eight models including Bard) performed better than the open‐source ones. Claude‐Pro and GPT‐4 models emerged as the top performers, predicting more genes correctly and having fewer false positives than the other models. The Claude‐Pro model topped the list with nine matched genes, FGFR1, PARD3, CDH3, CTNNB1, CDH1, CDC42, EP300, ACTB, and CTNND1, with weights 2, 2, 5, 4, 5, 3, 3, 3, and 4, respectively, including a Jaccard similarity of 0.450. There were only six unmatched genes. GPT‐4 matched eight genes, FGFR1, PARD3, CDH3, CTNNB1, CDH1, CDC42, ACTB, and CTNND1, with weights 2, 2, 5, 4, 5, 3, 3, and 4, respectively, with seven unmatched genes. This achieved a Jaccard similarity of 0.381.

In other API‐based models, Claude identified four genes, CDH1, CTNNA, CDH3, and CTNNB1, with weights 5, 3, 4, and 3, respectively. They achieved a Jaccard similarity of 0.167, leaving 11 genes unmatched. Claude‐Instant improved slightly, matching five genes, CTNNA, CDH3, CTNNB1, CDH1, and CTNND1, with weights of 2, 5, 4, 5, and 1, respectively. It had a Jaccard similarity of 0.208 and 10 unmatched genes. Cohere outperformed the previous two by identifying six genes, PARD3, CDH3, CTNNB1, CTNND1, CDH1, CTNNA, and ACTN4, with weights of 3, 4, 2, 4, 2, and 3, respectively. This was accompanied by a Jaccard similarity of 0.261, leaving nine genes unmatched. In contrast, GPT‐3.5 matched four genes, CTNNB1, CDH1, CDC42, and CTNND1, with weights 3, 5, 2, and 2, respectively, resulting in a Jaccard similarity of 0.167 and 11 unmatched genes.

In the open‐source category, the standout model was less clear, with several models displaying similar levels of performance. Codellama‐34 matched four genes, CDH3, CTNNB1, CDH1, and CTNND1, with weights 3, 2, 3, and 2, respectively. These genes yielded a Jaccard similarity of 0.167 and left 11 genes unmatched. Wizardlm_70 identified only two genes, CTNNB1 and CDH1, with equal weights of 3 each, resulting in a lower Jaccard similarity of 0.091 and 13 unmatched genes. The Falcon‐180b model matched five genes, CDH3, CTNNB1, CDH1, CDC42, and CTNND1, with weights of 3, 2, 3, 2, and 2, respectively. This achieved a Jaccard similarity of 0.208, leaving 10 genes unmatched. Mistral‐7b matched four genes, FGFR1, CDH3, CTNNB1, and CDH1, with weights 1, 2, 2, and 2, respectively. It yielded a Jaccard similarity of 0.174 and 10 unmatched genes. The Vicuna series offered varied results, and the best was Vicuna‐7b, with a Jaccard similarity of 0.217.

It is worth attention to the similarities and differences among 21 LLMs, as shown in a more detailed study with 10 runs for each LLM (Table S7). This indicates that even with more runs, a significant portion of the genes in the adherens junctions still cannot be predicted by any LLM, suggesting a potential ceiling for improving the recall rate by using ensemble learning [20] to combine multiple LLMs or by running many times to achieve better results. On the other hand, the ensemble learning strategy may work better to improve the precision rate. As shown in Table 2, the false positive genes are often predicted by one or a few LLMs. Using the best model in this prediction, Claude‐Pro (as an example), its false positive predictions, TJP1, ACTG1, CREBBP, AFDN, SRC, and VCL, were only predicted by 2, 1, 1, 1, 1, and 12 LLMs, respectively. Except for VCL, other false positive genes may be removed using an ensemble learning approach.

**FIGURE 4:**
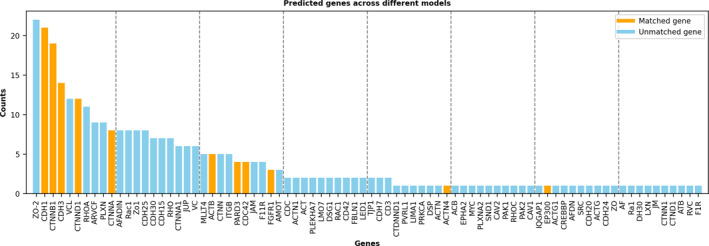
An ensemble learning analysis using all 21 LLMs for gene prediction in adherens junctions. All the predicted genes (80 in total) by the 21 LLMs are sorted by the number of predictions. Each model predicted five times, and hence, the maximum frequency is 105. Orange bars indicate true‐positive genes and purple bars show false‐positive genes. LLMs, large‐scale language models.

We conducted a simple ensemble learning test, as shown in Figure 4. The 21 LLMs predicted 80 genes in total, which are sorted by the number of the predictions in the figure. Interestingly, the top gene, ZO‐2, was not in adherens junctions according to the KEGG annotation (hence labeled as a false positive), but it is annotated as “Plays a role in tight junctions and adherens junctions (By similarity)” in UniProt [21]. Among the top six genes, four of them are in the ground truth, yielding a precision of 4/6 = 0.6667, which is very high. Out of the 80 genes, 11 of them are true positives, that is, the maximum recall that the ensemble learning model achieved is 11/14 = 0.7857, which is higher than any LLM’s recall in Table 2. Hence, there may be some values using ensemble learning to get the most confident genes or to explore more possible genes.

#### 2.2.2 Tight junction

Figure S1 comprehensively evaluates 21 LLMs in predicting genes linked to the Tight junction complex [22], a crucial component in cellular adhesion and signal transduction. Furthermore, Table S8 shows predicted genes that are not present in Tight junction. GPT‐4 and Claude‐Pro again emerged as the most proficient, both with a Jaccard similarity of 0.261 and predicting the same subset of genes correctly, closely followed by Falcon‐180b and llama2‐7b, both with a Jaccard similarity of 0.227. The other models ranged from moderate to low effectiveness in this task. Within the API‐based models, Cohere achieved a good Jaccard similarity of 0.208. Claude and Claude Instant had Jaccard similarities of 0.154 and 0.160, respectively. Models like Palm and Bard lagged, matching fewer genes and thus attained lower similarity scores. In the open‐source category, models like Codellama‐34 and Wizardlm‐70b exhibited moderate performance. The Mistral_7, Chatglm2‐6b, and the Vicuna series demonstrated varied outcomes, with Vicuna‐7b and Vicuna‐13b reaching a similarity score of 0.182. Qwen 14b fell slightly short in this regard.

#### 2.2.3 Gap junction

Figure S2 presents an analysis of 21 LLM capabilities in predicting genes associated with the Gap Junction complex, a pivotal element in cellular cohesion and signal transduction. Among these, GPT‐4 and Claude‐Pro were again identified as the most adept models, with similar performance. Other models’ relative performance was similar to that of tight junction. Table S9 complements these insights by examining the unmatched genes.

#### 2.2.4 Cellular senescence function

Figure S3 extensively evaluates the 21 LLMs in predicting genes linked to cellular senescence [23], another critical component in cellular communication and adhesion. GPT‐4 and Claude‐Pro again led in proficiency, but Bard tied their performance. They all have a Jaccard similarity of 0.250. Interestingly, GPT‐4 and Claude‐Pro predicted the same subset of six genes correctly, but Bard shared only five of them and predicted a different gene correctly, which provided some diversity. The performance of the remaining models varied from moderate to low. Table S10 shows the unmatched genes.

#### 2.2.5 Phagosome function

Figure S4 presents an extensive analysis of 21 LLM abilities to predict genes relevant to the phagosome function complex. This complex plays a significant role in cellular adhesion and signal transduction. GPT‐4 stood out for its accuracy, closely followed by Falcon‐180b. After these models, Claude‐Pro and llama2‐7b tied. Table S11 shows the unmatched genes.

#### 2.2.6 Proteoglycans in cancer

Figure S5 evaluates the 21 LLMs' accuracy in predicting genes associated with the proteoglycans in the cancer complex, a vital element in cellular adhesion and signal transmission. GPT‐4 and Claude‐Pro were again the most proficient, followed closely by Falcon‐180b and llama2‐7b. Table S12 shows the unmatched genes.

#### 2.2.7 Autoimmune thyroid disease

Figure S6 provides an evaluation of the 21 LLMs in terms of their capacity in predicting genes relevant to the autoimmune thyroid disease complex, a crucial aspect of cellular cohesion and communication. GPT‐4 and Claude‐Pro were identified as the most accurate, pursued by Falcon‐180b. Table S13 shows the unmatched genes.

#### 2.2.8 Overall evaluations

**FIGURE 5:**
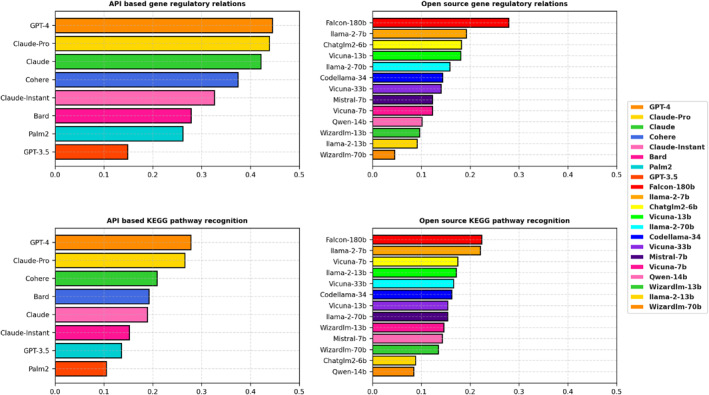
Comparative performance of 21 LLMs in gene regulatory relation prediction and KEGG pathway component recognition, categorized as API‐based and open‐source models. The upper two graphs are for regulatory relation prediction, assessed by the F1 score. The bottom two graphs are for KEGG pathway component recognition, assessed by Jaccard similarity. API, application programming interface; KEGG, Kyoto Encyclopedia of Genes and Genomes; LLMs, large‐scale language models.

Combining all the results in the previous section, we compared the overall performance of the 21 LLMs in Figure 5, dividing them into two distinct categories: API‐based and open‐source. Detailed performance values can be found in Table S14. This thorough assessment gauges their efficacy in predicting gene regulatory relations and identifying KEGG pathway components, a crucial aspect in interpreting complex biological data.

API‐based models GPT‐4 and Claude‐Pro clearly dominate the performance among 21 LLMs. In open‐source models, Falcon‐180b led the pack, outperforming its counterparts in both gene regulatory relations (0.2787, rank 1) and KEGG pathway recognition (0.2237, rank 1). This underscores its potential as a comprehensive tool in bioinformatics studies. Llama2‐7b also displayed a strong performance, ranking second in both gene regulatory relations (0.1923) and KEGG pathway recognition (0.2207). Codellama‐34b and Chatglm2‐6b gave robust performances in gene regulatory relations, scoring 0.1821 and 0.1435, respectively. The Vicuna and Llama2 series exhibited varying levels of performance. Notably, Vicuna‐7b, despite ranking ninth in gene regulatory relations, showed a specific strength in KEGG pathway recognition, ranking fourth. Qwen‐14b ranked the lowest in both gene regulatory relations (0.0100) and KEGG pathway recognition (0.0842), which revealed its challenges in interpreting complex biological data.

Overall, this evaluation sheds light on the diverse capabilities of the computational models, with certain models like GPT‐4 and Claude‐Pro showcasing high accuracy, and others such as Qwen‐14b exhibiting lower effectiveness. The variation in gene prediction confidence across different models underscores the importance of selecting the right computational tools for specific tasks in biological research.

## 3 DISCUSSION

In biomedical research, LLMs have been used to extract and analyze complex biological data. Researchers often use LLMs, especially GPT, to help research biomedical problems. However, to our knowledge, no systematic assessment has been conducted on the effectiveness of LLM retrieval of biomedical knowledge. This study comprehensively evaluated the efficacy of 21 LLMs, both API‐based and open‐source, in predicting gene regulatory relations and KEGG pathway components. A critical aspect of our research is the use of the ground truth in objective benchmarking. By employing metrics like accuracy, precision, recall, the F1 score, and the Jaccard similarity index, we have provided benchmarks and quantifiable comparisons of these models against established ground truths.

A notable finding from this study is the disparity in performance among different models. API‐based models like Claude‐Pro and GPT‐4 have demonstrated exceptional capabilities, potentially due to their large model sizes and inclusion of training text specific to biomedical research [16]. Building upon the findings discussed in our analysis (see Figure 1, Figures S1–S6), we observed a broad spectrum of performances among LLMs in their capacity in predicting gene regulatory relations and recognizing components within the KEGG pathway. On the one hand, many similar results can be found among various LLMs. For example, the similarities in training methodologies and datasets between top‐performing models such as GPT‐4 and Claude‐Pro result in a significant overlap in their predictions, as illustrated in our comparative analysis. This study revealed the limited capacities of ensemble learning by using multiple LLMs to retrieve more ground‐truth genes or relationships. On the other hand, combining all 21 LLMs can help exclude some false positives among top predictions, as false‐positive genes are often less likely to overlap than true positives among different LLMs. Hence, ensemble learning of multiple LLMs may improve prediction precision, that is, reduce hallucinations.

While LLMs offer significant potential in biomedical research, their deployment must be carefully selected and managed, focusing on enhancing accuracy and mitigating biases. The performance disparity among the evaluated models, particularly between the leading API‐based models like Claude‐Pro and GPT4, and the best‐performing open‐source models Falcon‐180b and llama2‐7b, underscores the significance of model selection tailored to the specific demands of biomedical tasks. Our study also identified LLM limitations that need to be addressed. The accuracy and specificity of these models, particularly in identifying complex biomedical terms, require further improvement. This could potentially be achieved through more specialized training on targeted datasets [24]. Integrating LLMs with existing bioinformatics tools and databases could lead to a more comprehensive approach to gene function and pathway analysis [25]. Additionally, the continual evolution of LLM algorithms and their adaptation to specific biomedical applications are crucial [26].

Prompt design in LLMs is crucial for model effectiveness. Precise prompts enhance output accuracy in tasks like gene regulatory relationship identification. We addressed LLMs’ stochastic nature by using a temperature setting of 0.9, encouraging diverse responses for detailed gene interaction insights, which resulted in informative prediction. This approach underscores the importance of operational considerations in LLM deployment for biomedical knowledge extraction, highlighting areas for future research optimization.

There are some limitations of this study as well. The study’s focus on a specific set of 200 genes and seven pathways presents a limitation in terms of the breadth of the research. While this selection allows for an in‐depth analysis of these genes, it may not fully capture the diversity and complexity inherent in biomedical research at large. The findings derived from these specific gene and pathway sets might not be representative of other gene sets, which could exhibit different patterns or interactions. This limitation points to the need for caution in generalizing this study’s results to the broader field of biomedicine. The use of different prompts in the study introduces a variable that could affect the consistency and reliability of the results. Since LLMs’ responses can vary significantly based on the input prompts, the diversity in the prompts used might lead to varied responses, making it challenging to accurately assess the overall performance and capabilities of the LLMs under different prompts. This variability could skew the study’s findings, as the LLMs might perform better or worse depending on the prompts used.

In summary, our study underscores the great potential of LLMs in biomedical knowledge mining, including gene regulatory network analysis and pathway mapping. Integrating LLMs into the biomedical research ecosystem is promising in transforming how complex biological data is analyzed and interpreted, thereby accelerating scientific discoveries and innovations in biology and healthcare. This necessitates continuous evolution and refinement of LLM algorithms, ensuring their relevance and efficacy in the rapidly advancing field of biomedical research.

## 4 MATERIALS AND METHODS

Figure 1 depicts the workflow in the results section for using LLMs to analyze biomedical data. This study extensively utilized the KEGG pathway database as the primary source for establishing the ground truth. We first analyzed gene regulatory relationships, with an emphasis on processes including activation, inhibition, and phosphorylation. To this end, we established interactions from the KEGG pathway. Then we selected seven crucial biomedical terms within the KEGG pathway. Prompts were crafted and applied to each LLM to extract pertinent data. We then comprehensively evaluated the performance of these models using statistical measures, such as accuracy, precision, recall, and F1 scores, along with the Jaccard similarity index for matching genes.

### 4.1 Evaluating gene regulatory relations

We employed the KEGG Markup Language (KGML) for effective data extraction and integration [27]. A key element of our methodology was the selection of 200 gene pairs from diverse pathway maps, representing three types of gene interactions: activation, inhibition, and phosphorylation, thereby providing a comprehensive framework for our experiments. These 200 gene pairs cover 80 instances each for activations and inhibitions, and 40 for phosphorylation. The complete list of 200 gene pairs can be found in Table S1.

Creating suitable prompts is crucial to fully leveraging the power of LLMs, particularly for extracting in‐depth knowledge about gene regulatory dynamics. Our strategy stresses the importance of developing prompts that are both precise and concise, demonstrating their success through few‐shot and role‐prompting techniques [28]. These approaches are designed to solicit highly specific information from LLMs. Our early observations indicated a significant variance in prompt effectiveness. Some prompts excelled with API‐driven models, but their performance waned with open‐source variants. After comprehensive testing and assessment, we identified a definitive prompt that consistently achieved robust outcomes across both API and open‐source models. An example of such a prompt, devised for computational biologists, was: “Identify the interaction between {gene1} and {gene2} within the KEGG Pathway Database.” This prompt focuses sharply on straightforward classifications like activation, inhibition, or phosphorylation, emphasizing the importance of clarifying gene relationship directions. It is crafted to guarantee that the responses are direct and strictly relevant, avoiding any irrelevant details. This strategy for prompt creation is designed for broad application across various LLM platforms and eliminates the need for model‐specific fine‐tuning. Table S2 provides some prompts we tested and the final one we selected.

### 4.2 Evaluations of KEGG pathway recognition

We focused on identifying seven key biomedical terms within the KEGG pathway framework. These terms are Adherens Junction, Tight Junction, GAP Junction, Cellular Senescence, Phagosome, Proteoglycans in Cancer, and Autoimmune Thyroid Disease. The selection of the seven KEGG terms in the manuscript was based on their diverse representation of key biomedical areas. This would allow for a thorough assessment of LLM predictive accuracies and their utilities in gene regulatory relationship identification within complex biological systems. The comprehensive ground truth data for all the examined biomedical terms is detailed in Figures 1 and S1–S6. The prompts we applied are shown in Table S6. We employed 21 different LLMs to assess their accuracy in identifying these specific biomedical terms. Our evaluation was based on a detailed comparison with verified data related to these terms. Our analysis included not only instances where the models successfully identified the correct gene associations but also cases where they failed to match genes. To improve the reliability of our assessments, we repeated one query five times, assigning a weighted significance to each gene based on its occurrence.

### 4.3 Large language models

We investigated the capabilities of advanced LLMs, focusing on both API‐based and open‐source models, including smaller, specialized models tailored for the biomedical domain. Our goal was to assess their proficiency in various biological tasks, with a particular emphasis on pathway knowledge and gene regulatory relationships [29]. The technical specifications of these models are detailed in Table 3. For API‐based models, the results were obtained through programmatic API calls instead of web interfaces; for open‐source models, we installed them on our machines and performed the predictions locally.

**TABLE 3:** Specifications of the 21 LLMs used in this study.

Type		Model	Release date	Developed by	Parameters	Context length	Feature
API model	ChatGPT	GPT‐4 [30]	14 March 2023	OpenAI	Not disclosed	8192 tokens (initial), later 32,768 tokens	Text input only in the initial version
		GPT‐3.5‐turbo [31]	November 2023	OpenAI	Not specified	128k context window	Upgraded from 16k
	Claude	Claude [32]	March 2023	Anthropic	Not specified	Not specified	Improved performance, longer responses, accessible via API, improved in coding, math, and reasoning capabilities​
		Claude‐Pro [32]	July 2023	Anthropic	Not specified	Not specified	Premium version with enhanced usage
		Claude‐instant [33]	August 2023	Anthropic	Not specified	9k tokens (7000 words)/100k tokens (75,000 words)	Fastest model, strong in creative tasks
		PaLM‐2 [34]	2023	Google	14.7 billion—100 billion	Not specified	Advanced reasoning, coding, and mathematics
		Bard [35]	March 2023	Google	(Based on PaLM 2)	Not specified	Image capabilities, coding features, app integration
		Cohere [36]	June 2022	Cohere	52 billion	4k tokens	Generative large language model
Open‐ source	Wizardlm	Wizardlm‐70b [37]	August 2023	Meta	70 billion	4k	Transformer‐based language model fine‐tuned on AI‐evolved instructions
		Wizardlm‐13b [38]	July 2023	Llama using Evol instruct	Not specified	Not specified	Supports multi‐turn conversation, trained without alignment or moralizing
							Includes extended context, a chain of thought, and story‐telling. Requires strong hardware for best performance
	Vicuna	Vicuna‐7b [39]	March–April 2023	LMSYS	7 billion	2048 tokens	Open source chatbot, fine‐tuned from Llama
		Vicuna‐33b [39]	2023	LMSYS	33 billion	16k	Larger context window, improved accuracy
		Vicuna‐13b [39]	April 2023	LMSYS	13 billion	4k or 16k	Open source chatbot, fine‐tuned from Llama
	llama2‐	llama2‐13b‐chat [40]	2023	Meta	13 billion	4096 tokens	Part of Llama 2 series, trained on 2 trillion tokens
		llama2‐7b [40]	2023	Meta	7 billion	4096 tokens	Part of Llama 2 series, trained on 2 trillion tokens
		llama2‐70b [40]	2023	Meta	70 billion	4096 tokens	Part of Llama 2 series, trained on 2 trillion tokens
		Falcon‐180b [34, 41]	September 2023	Mistral AI	180 billion	Not specified	Largest openly available language model
		Mistral‐7b [42]	2023	Mistral AI	7.3 billion	8000 tokens	Powerful language model for its size
		Chatglm2‐6b [43]	June 2023	Tsinghua	6 billion	32k	Improved reasoning ability
		Codellama‐34 [44]	24 August 2023	Meta	34 billion	16k (up to 100k extrapolated)	State‐of‐the‐art LLM for coding
		Qwen‐14b [45]	July 2023	Quantum AI	14 billion	6000 words	Optimized for Quantum computing and data analysis

Abbreviation: LLMs, large‐scale language models.

### 4.4 Evaluations

Our analysis assessed the performance of various LLMs in predicting gene regulatory relationships or pathway components within KEGG pathways [46]. The evaluation metrics were calculated by comparing the ground truth data, which represents the actual gene relationships or pathway components, with the predictions made by each LLM. Here’s how each metric was contextualized:

Precision: In our study, precision quantifies the accuracy of the LLMs by correctly predicting gene regulatory relationships (or pathway components). It reflects the model’s ability to minimize false positives, ensuring that the relationships it predicts are indeed relevant and accurate:

$$P\text{recision}=\frac{\text{Number}\;\text{of}\;\text{correctly}\;\text{predicted}\;\text{gene}\;\text{relationships}}{\text{Number}\;\text{of}\;\text{gene}\;\text{relationships}\;\text{predicted}\;\text{by}\;\text{the}\;\text{model}}$$

Recall (Sensitivity): Recall measures the selected LLMs’ ability to identify all existing gene regulatory relationships or pathway components in the KEGG pathways. It ensures that the model captures as many true regulatory relationships (or pathway components) as possible, minimizing missed detections (false negatives):

$$\text{Recall}=\frac{\text{Number}\;\text{of}\;\text{correctly}\;\text{predicted}\;\text{gene}\;\text{relationships}}{\text{Number}\;\text{of}\;\text{actual}\;\text{gene}\;\text{regulatory}\;\text{relationships}}$$

F1 score: The F1 score balances the trade‐off between precision and recall. It provides a single metric that encapsulates the model’s overall performance in predicting gene regulatory relationships within KEGG pathways:

$$F1=2*\frac{\text{Precision}*\text{Recall}}{\text{Precision}+\text{Recall}}$$

Jaccard similarity: This metric was selected to evaluate LLM performance in recognizing gene components related to the KEGG Pathways [47]. The central objective of this evaluation was to analyze the gene overlaps by comparing the genes predicted by an LLM to the actual, known genes—a set we refer to as the “ground truth.” The Jaccard similarity measures the proportion of common genes to the total unique genes identified by the model predictions and the actual data:

$$\text{Jaccard}\;\text{similairty}=\frac{\text{Number}\;\text{of}\;\text{common}\;\text{genes}\;}{\text{Total}\;\text{number}\;\text{of}\;\text{genes}\;\text{in}\;\text{both}\;\text{sets}\;}$$

## AUTHOR CONTRIBUTIONS

**Muhammad Azam**: Data curation; formal analysis; methodology; software; visualization; writing – original draft; writing – review & editing. **Yibo Chen**: Data curation; formal analysis; software; writing – review & editing. **Micheal Olaolu Arowolo**: Data curation; validation; writing – review & editing. **Haowang Liu**: Formal analysis; software. **Mihail Popescu**: Funding acquisition; investigation; resources; writing – review & editing. **Dong Xu**: Conceptualization; funding acquisition; investigation; methodology; project administration; resources; supervision; writing – review & editing.

## CONFLICT OF INTEREST STATEMENT

The authors Muhammad Azam, Yibo Chen, Micheal Olaolu Arowolo, Haowang Liu, Mihail Popescu, and Dong Xu state no conflict of interest.

## ETHICS STATEMENT

This article does not contain any studies with human or animal materials performed by any of the authors.

## Supporting information

Supporting Information S1

## Data Availability

The data structure concerning gene pathways and biomedical terms, as developed and analyzed in our study, can be found on the KEGG website and in the Supplemental File.
