# Supplementary material for: A comprehensive evaluation of large language models in mining gene relations and pathway knowledge
Source: Quant Biol. 2024 Jun 21;12(4):360–74. doi: 10.1002/qub2.57 (PMC11446478; doi:10.1002/qub2.57)
Supplement: Supplementary file 1 — Supporting Information S1 [file QUB2-12-360-s001.docx]

Detection of genes in various model predictions for Tight junction

**
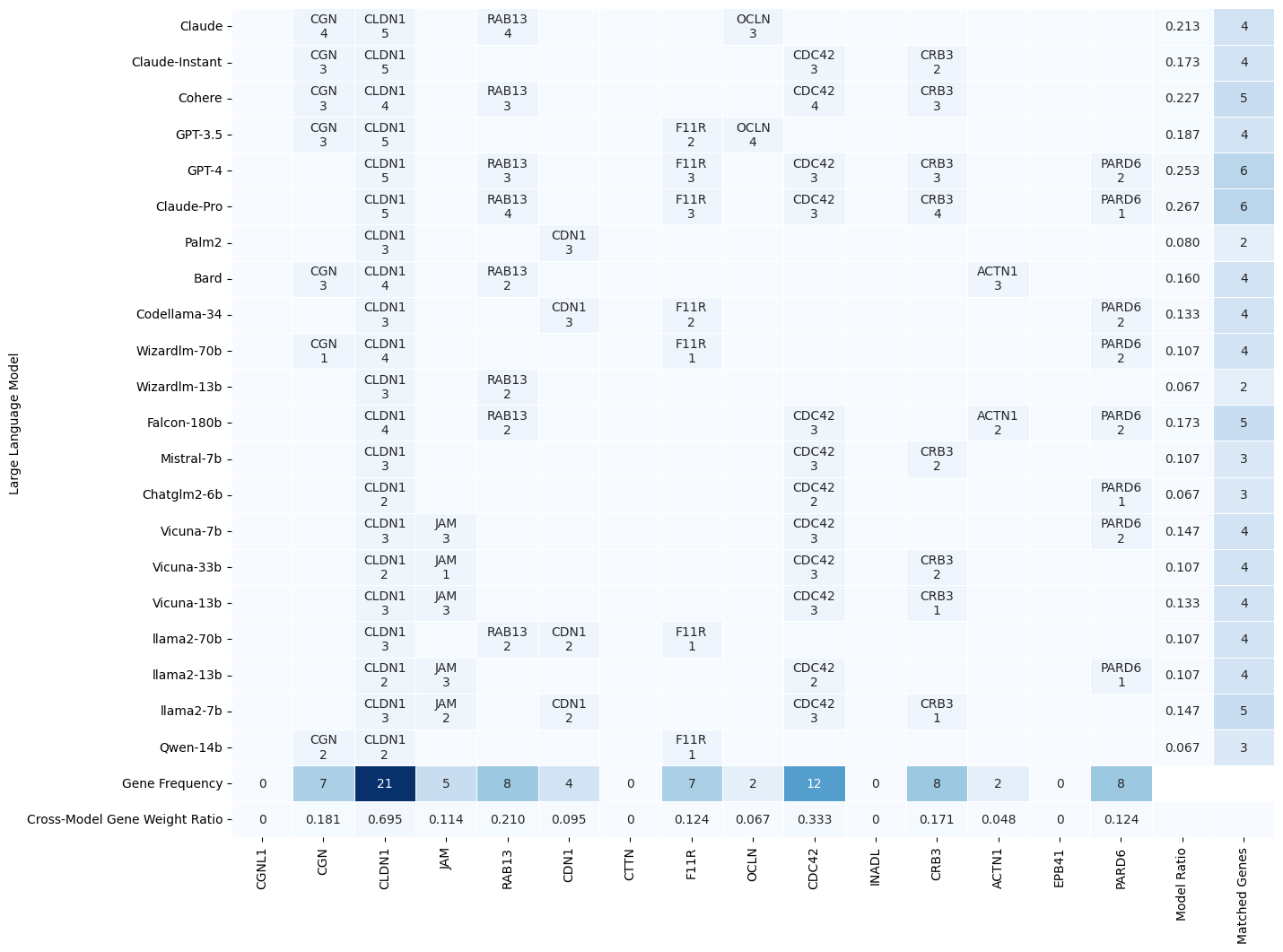
**

Tight junction pathway genes

**(A)**

Jaccard similarity to Tight junction **
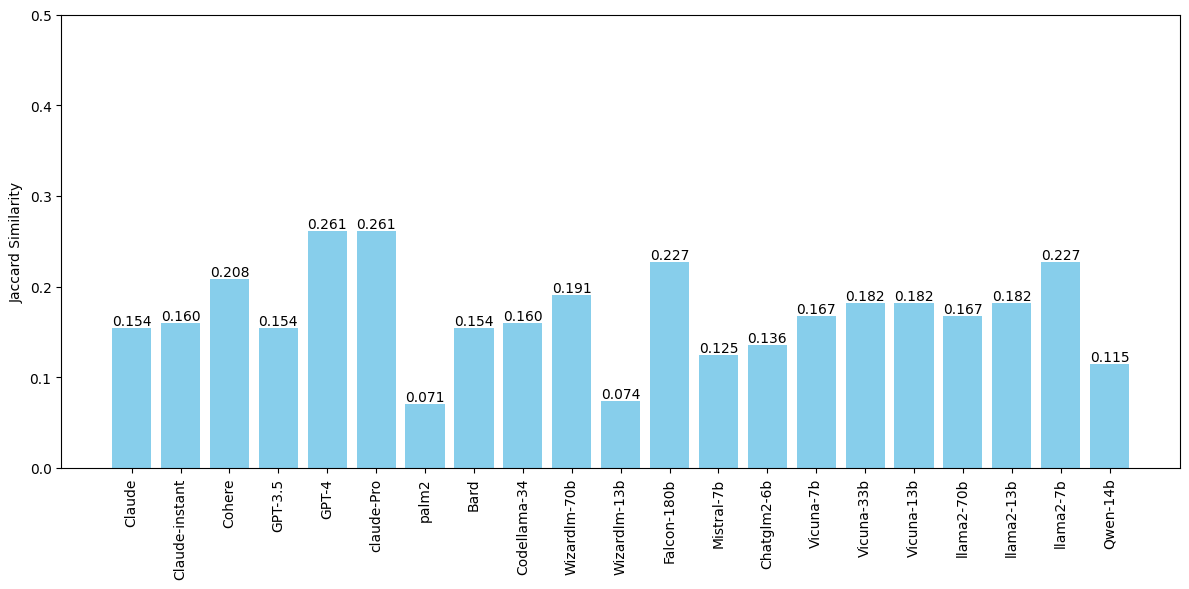
**

LLM model

**(B)**

**Figure S1:** Gene predictions for tight junctions. **(A)** displays prediction accuracy and confidence scores from the 21 API-based and open-source models. The bottom two rows show the frequency of correct gene occurrence among models (one or more correct predictions are counted as success for the model and among all predictions). Cross-model gene weight ratio is the number of correct predictions divided by the number of all predictions, where each model has five predictions for a given gene. The two columns on the far right show the frequency of correct gene occurrence in all predictions (the number of correct predictions divided by the number of all predictions for the model, where each model has five predictions), and the number of correctly predicted genes (for a given model, one or more correct predictions from the model on the gene are counted as a success for that gene). **(B)** compares the models through Jaccard similarity scores, assessing their accuracy in matching gene sets specific to Tight Junctions.

Detection of genes in various model predictions for GPA junction


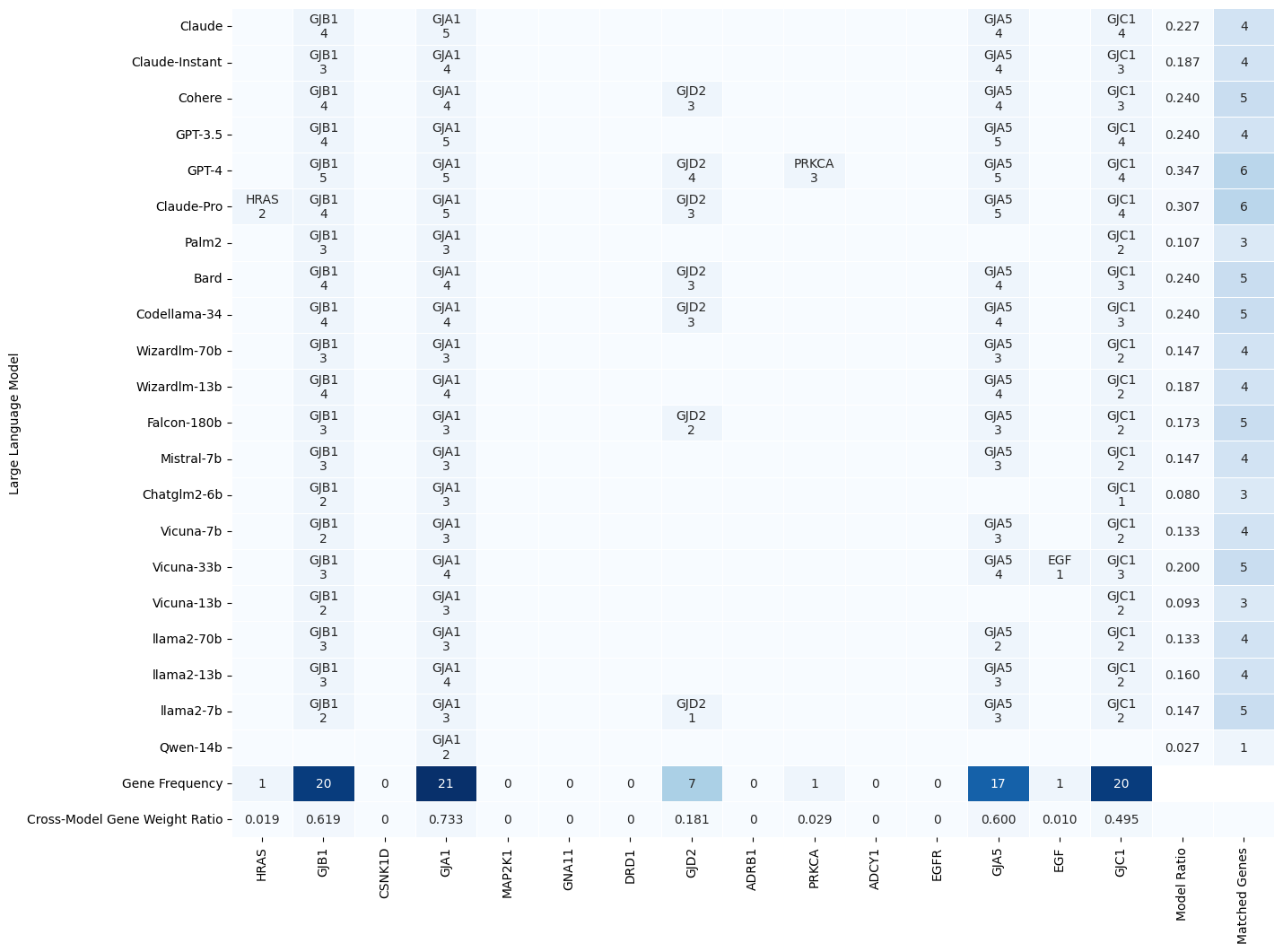


Gap junction pathway genes

**(A)**

Jaccard similarity to GAP junction
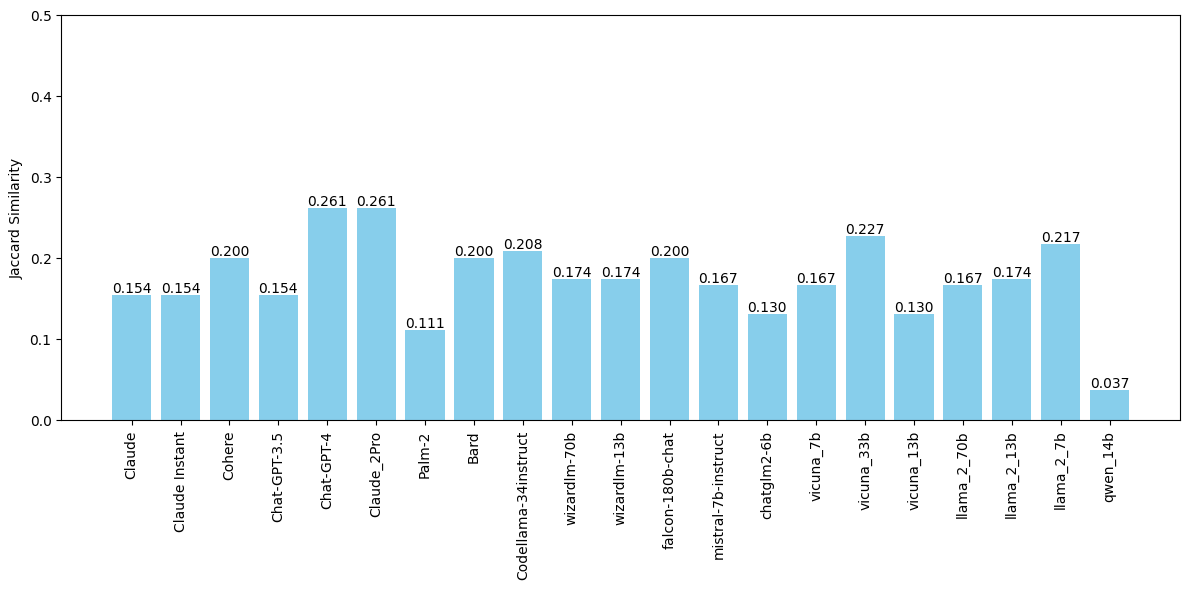


LLM model

**(B)**

**Figure S2:** Gene predictions for gap junctions. **(A)** displays prediction accuracy and confidence scores from the 21 API-based and open-source models. The bottom two rows show the frequency of correct gene occurrence among models. One or more correct predictions are counted as success for the model. Furthermore, among all predictions, the cross-model gene weight ratio is the number of correct predictions divided by the number of all predictions, wherein each model has five predictions for a given gene. The two columns on the far right show the frequency of correct gene occurrence in all predictions (the number of correct predictions divided by the number of all predictions for the model, where each model has five predictions), and the number of correctly predicted genes (for a given model, one or more correct predictions from the model on the gene are counted as a success for that gene). (**B)** compares the models through Jaccard similarity scores, assessing their accuracy in matching gene sets specific to gap junctions.

Detection of genes in various model predictions for Cellular Senescence

**
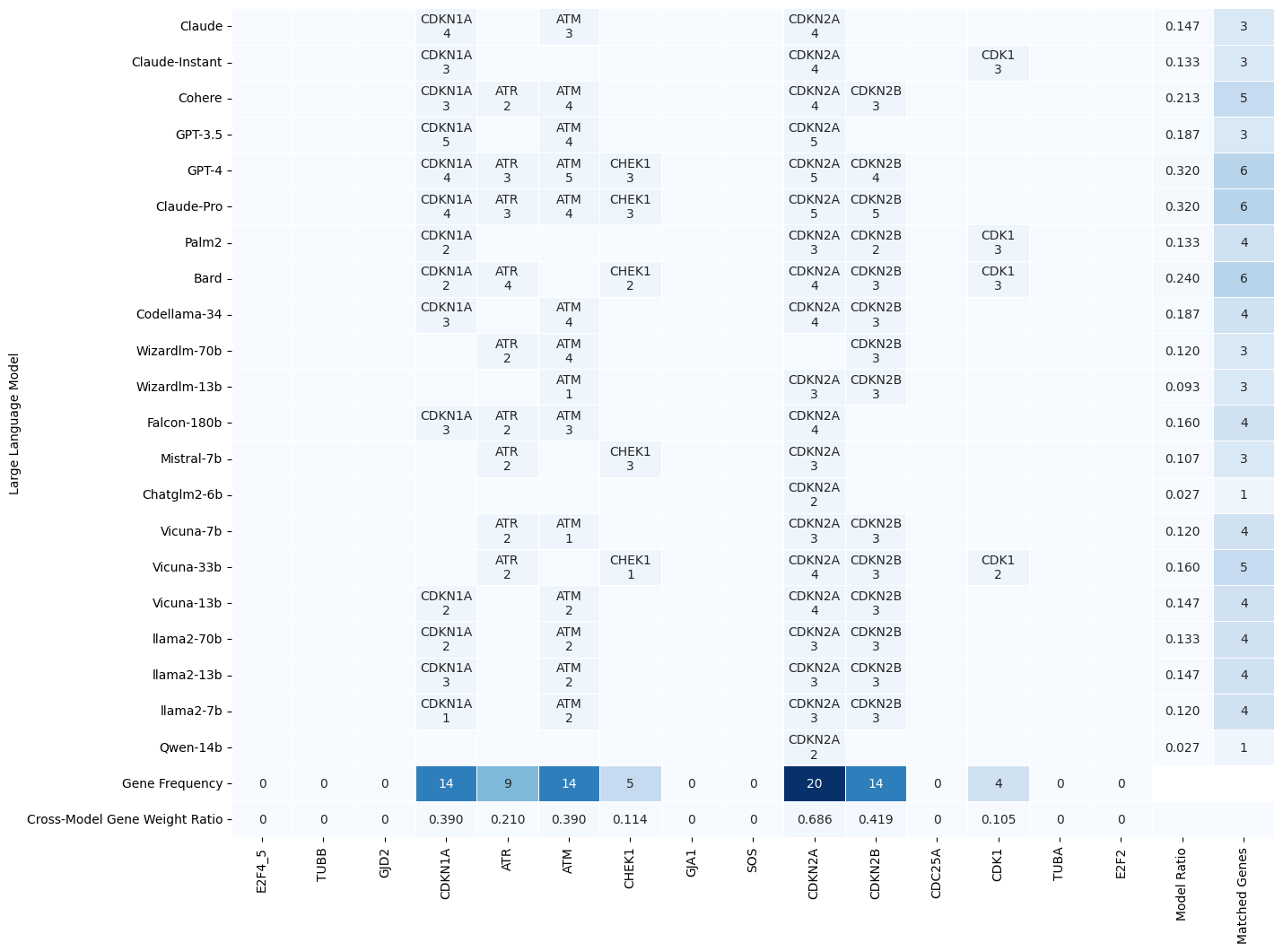
**

Cellular Senescence pathway genes

**(A)**

Jaccard similarity to Cellular Senescence


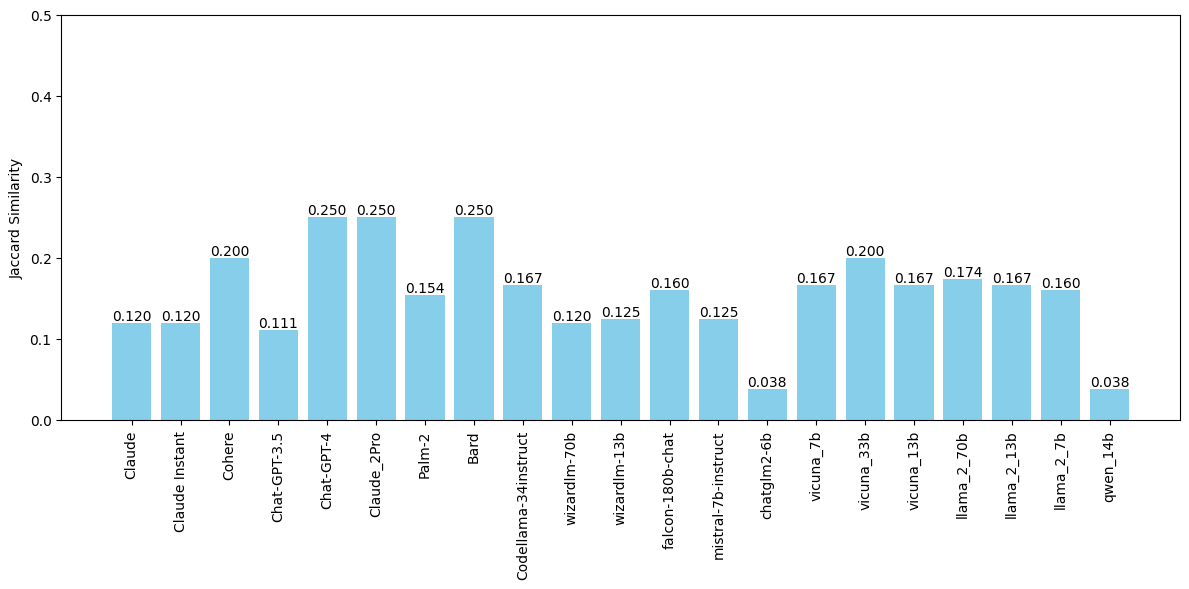


LLM model

**(B)**

**Figure S3:** Gene predictions for cellular senescence. **(A)** displays prediction accuracy and confidence scores from the 21 API-based and open-source models. The bottom two rows show the frequency of correct gene occurrence among models. One or more correct predictions are counted as success for the model and among all predictions. Cross-model gene weight ratio is the number of correct predictions divided by the number of all predictions, where each model has five predictions for a given gene. The two columns on the far right show the frequency of correct gene occurrence in all predictions (the number of correct predictions divided by the number of all predictions for the model, where each model has five predictions), and the number of correctly predicted genes (for a given model, one or more correct predictions from the model on the gene are counted as a success for that gene). **(B)** compares the models through Jaccard similarity scores, assessing their accuracy in matching gene sets specific to cellular senescence.

Detection of genes in various model predictions for Phagosome Function

**
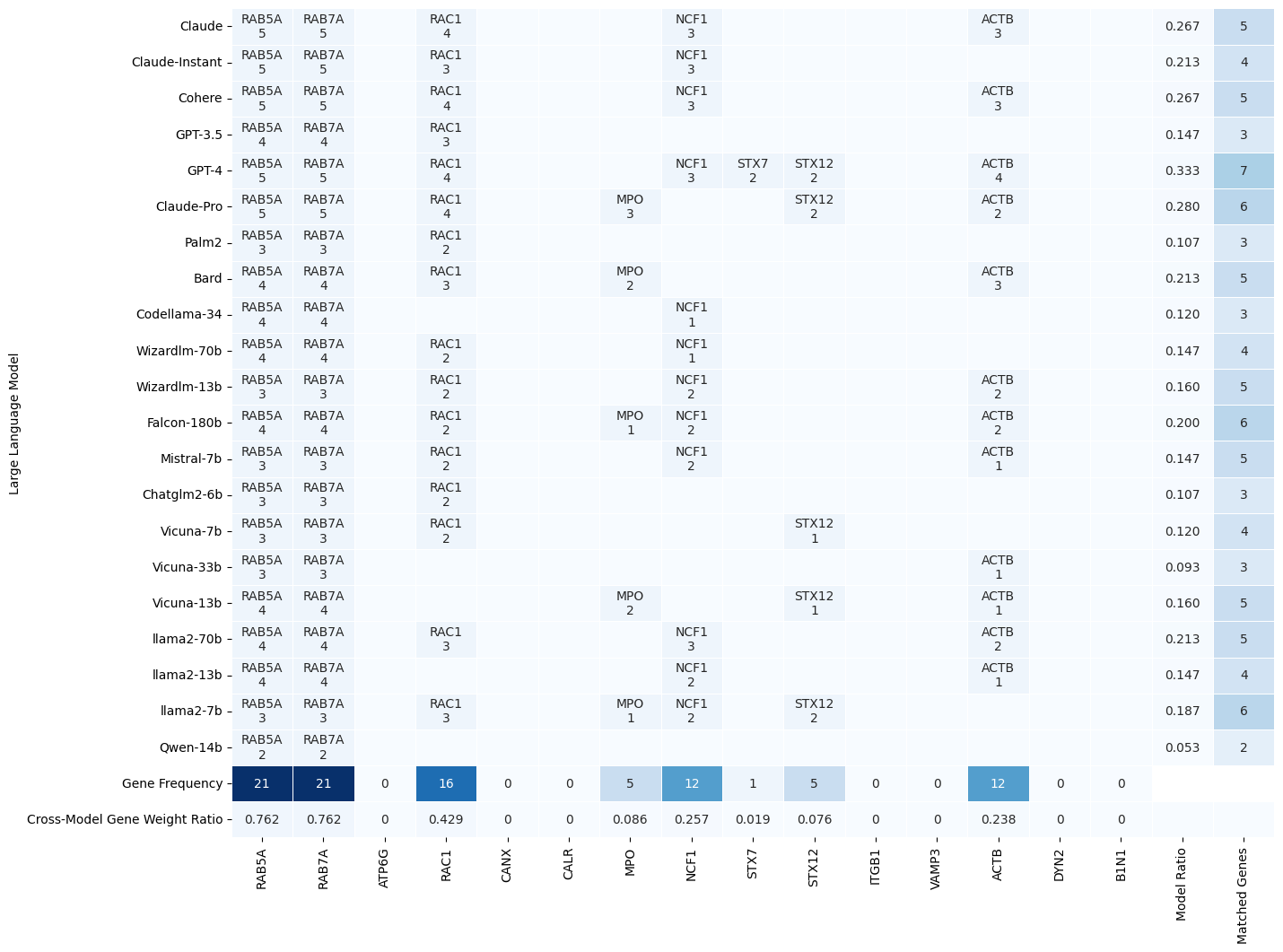
**

Phagosome Function pathway genes

**(A)**

Jaccard similarity to Phagosome Function

**
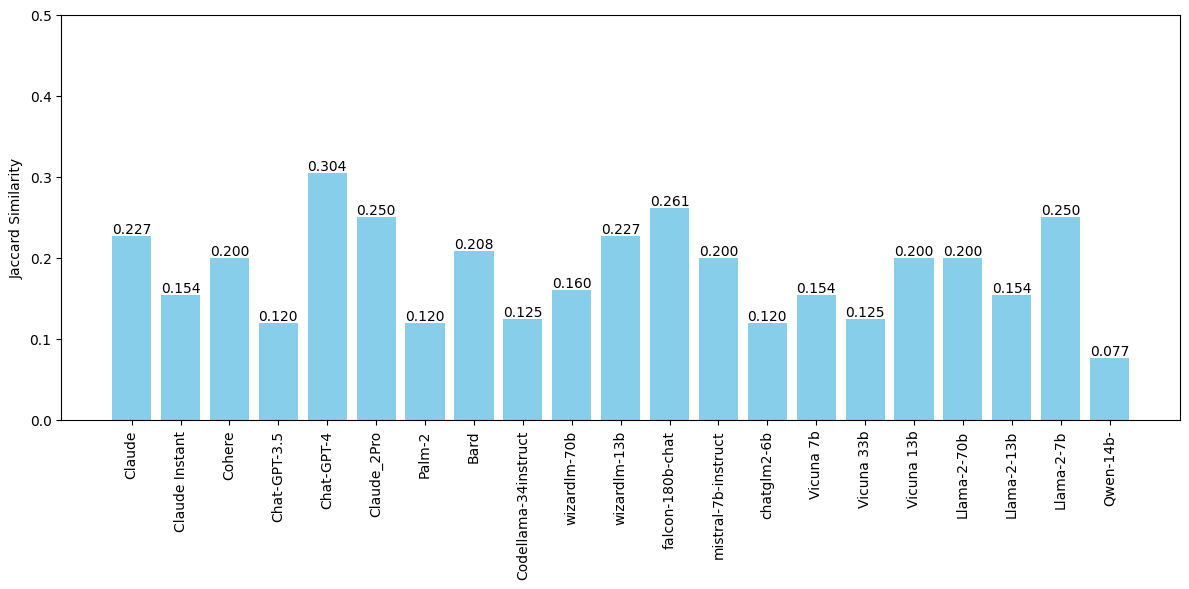
**

LLM model

**(B)**

**Figure S4:** Gene predictions for phagosome function. **(A)** displays prediction accuracy and confidence scores from the 21 API-based and open-source models. The bottom two rows show the frequency of correct gene occurrence among models. One or more correct predictions are counted as success for the model and among all predictions. Cross-model gene weight ratio is the number of correct predictions divided by the number of all predictions, where each model has five predictions for a given gene. The two columns on the far right show the frequency of correct gene occurrence in all predictions (the number of correct predictions divided by the number of all predictions for the model, where each model has five predictions), and the number of correctly predicted genes (for a given model, one or more correct predictions from the model on the gene are counted as a success for that gene). **(B)** compares the models through Jaccard similarity scores, assessing their accuracy in matching gene sets specific to phagosome function.

Detection of genes in various model predictions for Proteoglycans in cancer


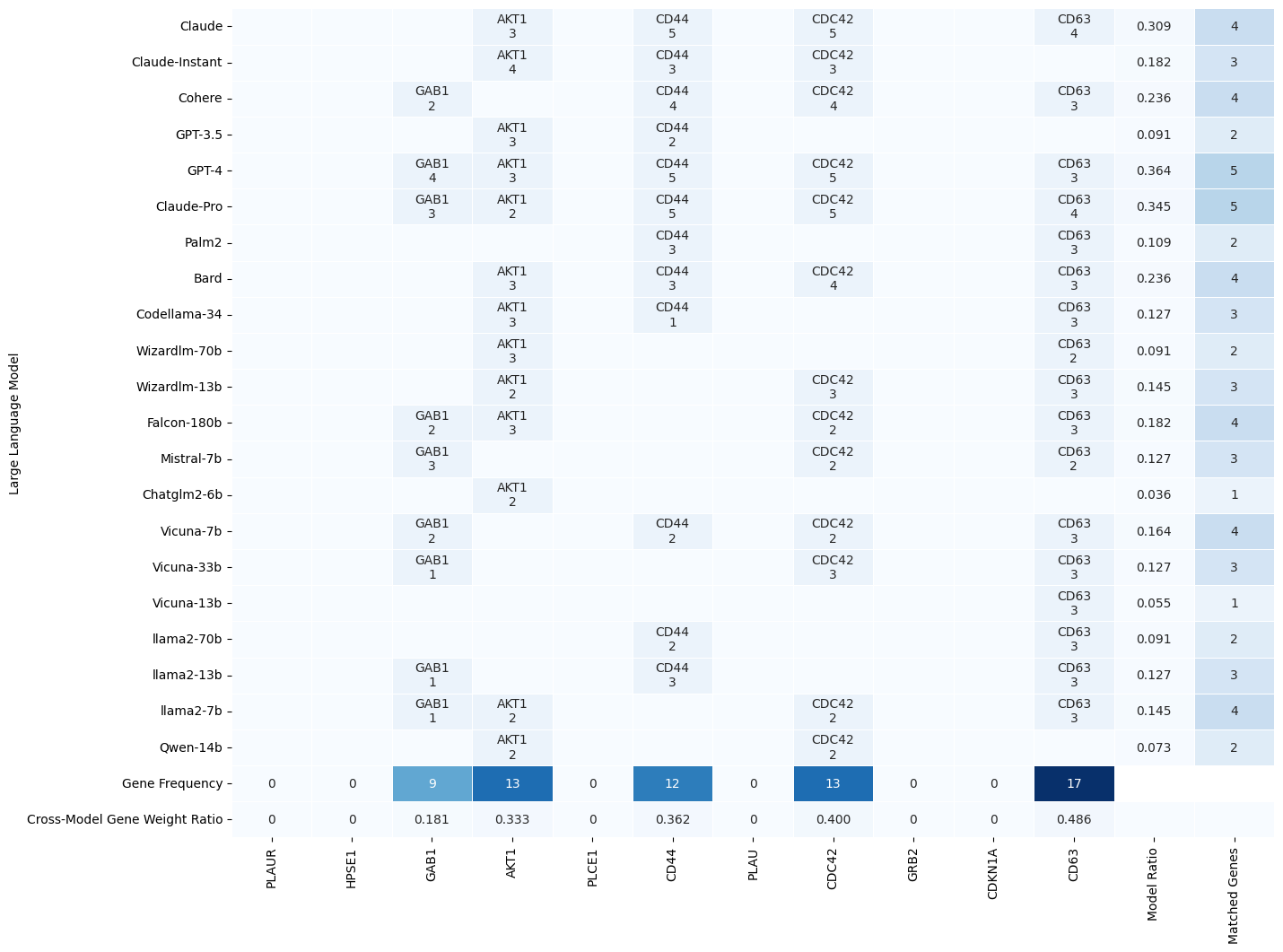


Proteoglycans in cancer pathway genes

**(A)**

Jaccard similarity to Proteoglycans in cancer

**
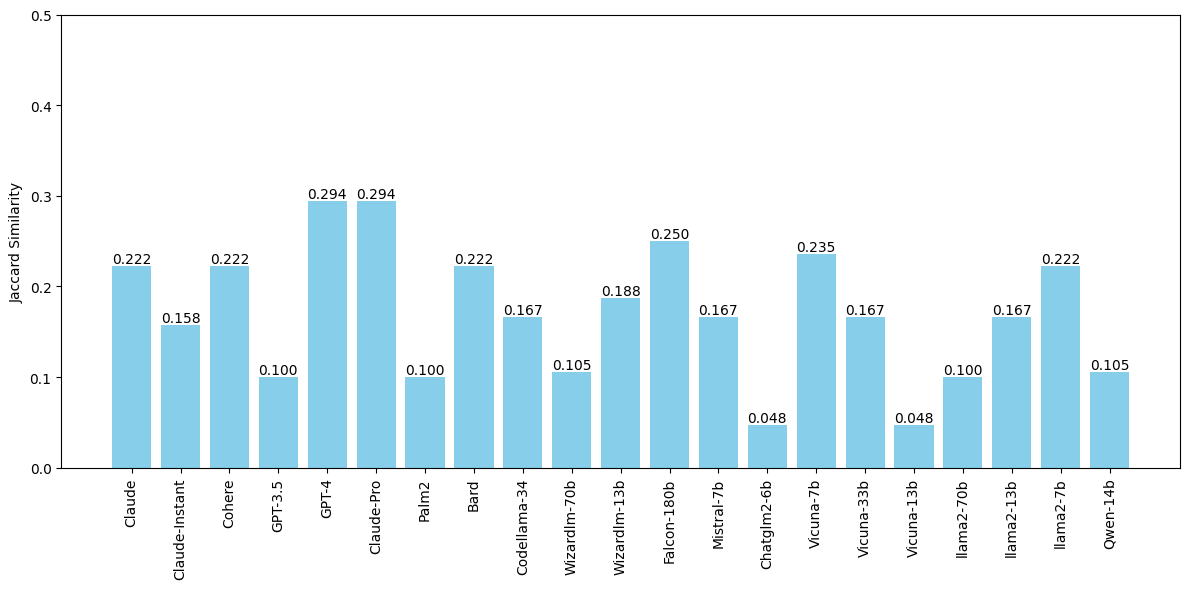
**

LLM model

**(B****)**

**Figure S5:** Gene predictions for proteoglycans in cancer. **(A)** displays prediction accuracy and confidence scores from the 21 API-based open-source models. The bottom two rows show the frequency of correct gene occurrence among models. One or more correct predictions are counted as success for the model. Moreover, among all predictions, the cross-model gene weight ratio is the number of correct predictions divided by the number of all predictions, where each model has five predictions for each given gene. The two columns on the far right show the frequency of correct gene occurrence in all predictions (the number of correct predictions divided by the number of all predictions for the model, where each model has five predictions), and the number of correctly predicted genes (for a given model, one or more correct predictions from the model on the gene are counted as a success for that gene). **(B)** compares the models through Jaccard similarity scores, assessing their accuracy in matching gene sets specific to proteoglycans in cancer.

Detection of genes in various model predictions for Autoimmune Thyroid Disease


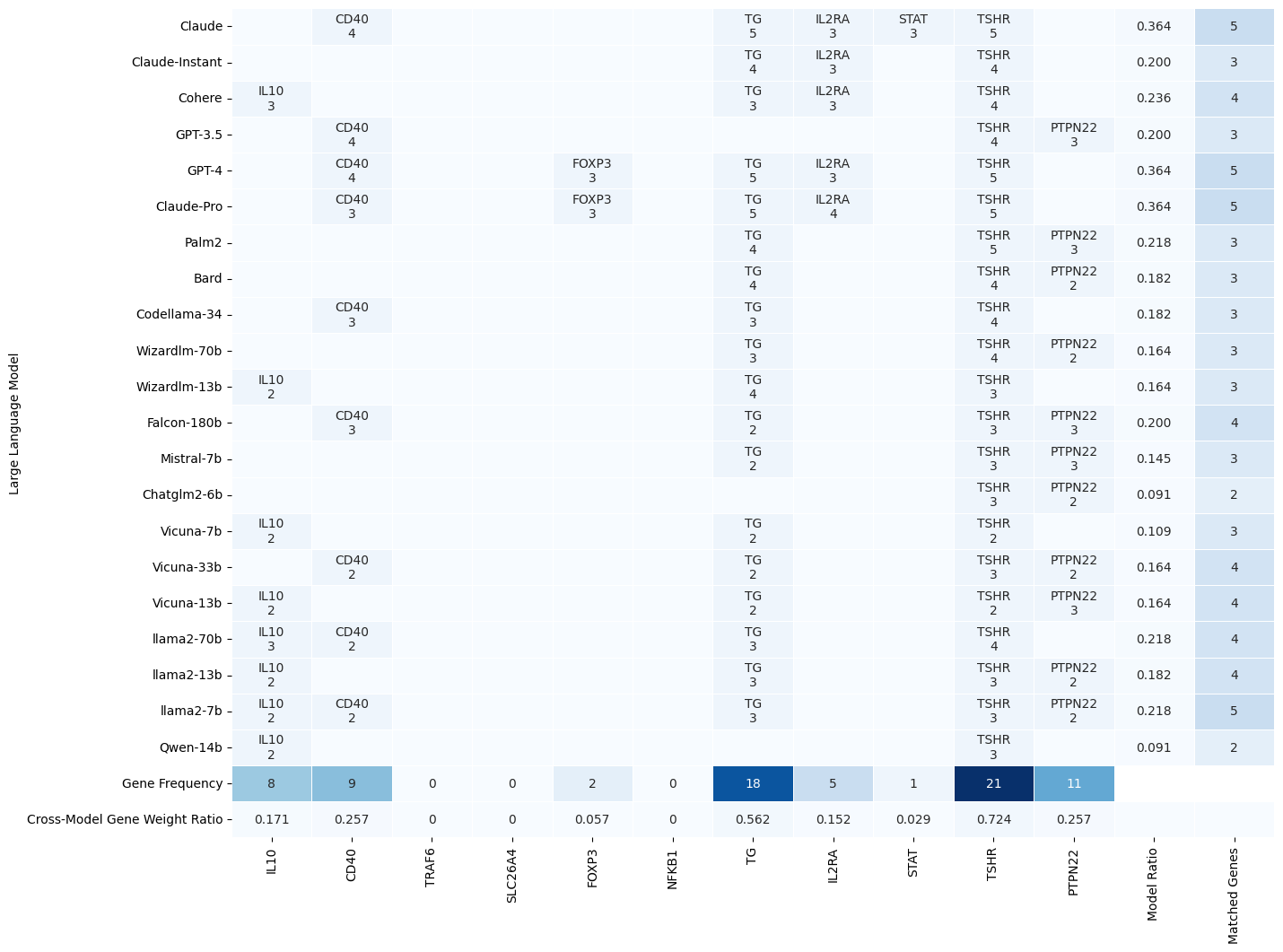


Autoimmune Thyroid Disease pathway genes

**(A)**

Jaccard similarity to Autoimmune Thyroid Disease
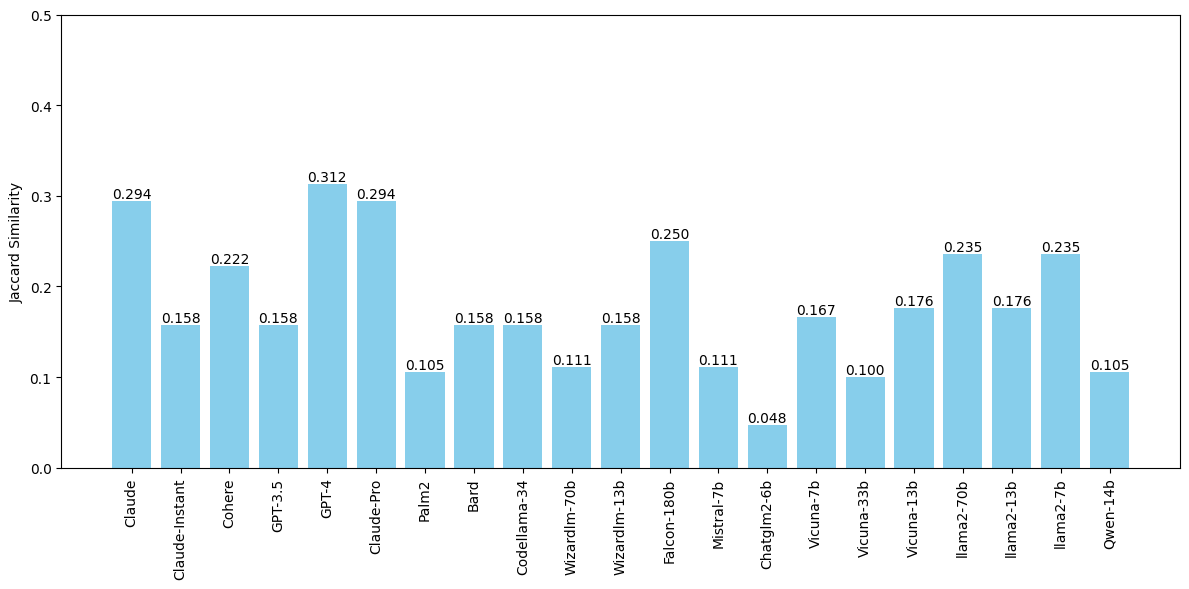


LLM model

**(B)**

**Figure S6:** Gene predictions for autoimmune thyroid disease. **(A)** displays prediction accuracy and confidence scores from the 21 API-based and open-source models. The bottom two rows show the frequency of correct gene occurrence among models where one or more correct predictions are counted as success for the model. Furthermore, among all predictions, the cross-model gene weight ratio is the number of correct predictions divided by the number of all predictions. Each model has five predictions for a given gene. The two columns on the far right show the frequency of correct gene occurrence in all predictions (the number of correct predictions divided by the number of all predictions for the model, where each model has five predictions), and the number of correctly predicted genes (for a given model, one or more correct predictions from the model on the gene are counted as a success for that gene). **(B)** compares the models through Jaccard similarity scores and assesses their accuracy in matching gene sets specific to autoimmune thyroid disease.

**Table S1:** The 200 gene triplets used in this study

| **Starter** | **Receiver** | **Status** |
| --- | --- | --- |
| IL17D | IL2RB | activation |
| OR4N2 | GNG13 | activation |
| IL12A | IL23R | activation |
| HSD17B10 | SLC25A5 | activation |
| NTRK2 | SHC3 | activation |
| IL6 | IFNGR2 | activation |
| GNG7 | PIK3R2 | activation |
| OR2T5 | GNB1 | activation |
| OR5AL1 | GNB1 | activation |
| OR14A2 | GNAL | activation |
| CCR5 | GNB3 | activation |
| LIMK1 | CASP1 | activation |
| IFNB1 | IL10RA | activation |
| IFNA4 | CNTFR | activation |
| IL21 | IL10RA | activation |
| IL7 | OSMR | activation |
| TLR6 | MAPK1 | activation |
| HLA-A | CD8B | activation |
| EPHA8 | ABL1 | activation |
| ITGB1 | PIK3R3 | activation |
| LAMC1 | CD44 | activation |
| GNG13 | NRAS | activation |
| ATG5 | MAP1LC3A | activation |
| IL3 | IL9R | activation |
| PSEN1 | CTNNB1 | activation |
| FGF20 | ERBB3 | activation |
| CDC42 | BUB1B-PAK6 | activation |
| INS | NR1H2 | activation |
| TRAF2 | PIK3R1 | activation |
| FGFR3 | PLCB3 | activation |
| IL2 | IL20RB | activation |
| ATP6V1F | LAMTOR2 | activation |
| SHC1 | SOS2 | activation |
| FGF22 | FGFR3 | activation |
| IL15 | IFNGR1 | activation |
| GNA13 | PLA2G4C | activation |
| OR3A2 | GNG7 | activation |
| OR4K1 | GNAL | activation |
| PRKG2 | KCNMB4 | activation |
| CCL23 | CXCR6 | activation |
| WNT7A | FZD9 | activation |
| GDF6 | BMPR1B | activation |
| PRL | IL23R | activation |
| HRAS | RPS6KA1 | activation |
| CARD9 | RIPK2 | activation |
| CCR9 | JAK3 | activation |
| ITGAM | PIK3R2 | activation |
| IL19 | IL4R | activation |
| KDR | SHC1 | activation |
| GNGT1 | MRAS | activation |
| CCL25 | CCR5 | activation |
| CXCL2 | XCR1 | activation |
| IGF1R | PLCG1 | activation |
| TCL1A | AKT1 | activation |
| TRAF2 | PIK3CD | activation |
| VIP | ADORA2A | activation |
| EPHB1 | RASA1 | activation |
| OR6C68 | GNG7 | activation |
| TSHB | CRHR2 | activation |
| JAK2 | PIK3CA | activation |
| GNG11 | RAC3 | activation |
| GNAQ | KRAS | activation |
| ABL1 | STAT5A | activation |
| PRKAR1B | LIPE | activation |
| GNG7 | PIK3R1 | activation |
| IL15 | IFNAR1 | activation |
| MAPK11 | CREB5 | activation |
| ATXN3L | ITPR3 | activation |
| INSR | PIK3R3 | activation |
| PRKACB | GNB4 | activation |
| GNG10 | PLCB4 | activation |
| OR5AP2 | GNAL | activation |
| IFNA8 | IFNAR1 | activation |
| GNG5 | MRAS | activation |
| MAPK11 | FOS | activation |
| TNFRSF11A | BTK | activation |
| SRC | CLEC7A | activation |
| OXT | CHRM2 | activation |
| GRM2 | GNG4 | activation |
| BMPR1B | SMAD9 | activation |
| GREM2 | GDF6 | inhibition |
| LEFTY2 | TGFB2 | inhibition |
| CTSW | XIAP | inhibition |
| PRKACB | OR8H3 | inhibition |
| PTPN5 | MAPK3 | inhibition |
| PPP2R3B | CACNG2 | inhibition |
| AKT1 | FOXO6 | inhibition |
| MIRLET7C | KRAS | inhibition |
| SNCA | NDUFB5 | inhibition |
| MIR103A2 | RPS6KA5 | inhibition |
| PRKACA | OR51A2 | inhibition |
| LRPAP1 | LRP1 | inhibition |
| PPP1R1B | PRKACG | inhibition |
| GRK3 | OR13C4 | inhibition |
| GNAI1 | CACNA1S | inhibition |
| MIR1-2 | FOXP1 | inhibition |
| PPP1R3C | CALML4 | inhibition |
| MIR29C | DNMT3A | inhibition |
| PRKACB | ND6 | inhibition |
| PSEN2 | ERN1 | inhibition |
| MIR15A | WNT3 | inhibition |
| MAPK3 | FOXO3 | inhibition |
| TBP | GTF2B | inhibition |
| MAPT | TUBB8 | inhibition |
| GRK2 | OR56A1 | inhibition |
| PRKACA | OR2F1 | inhibition |
| YWHAQ | BRAF | inhibition |
| PRKACA | OR4K2 | inhibition |
| PTPRJ | PTK2 | inhibition |
| PPP1CA | CREB1 | inhibition |
| MAPK8 | BCL2 | inhibition |
| PRKACA | RAF1 | inhibition |
| MIRLET7A2 | CDC25B | inhibition |
| ARRB2 | OR4E1 | inhibition |
| DUSP5 | MAPK9 | inhibition |
| CTBP1 | TCF7 | inhibition |
| SOCS1 | STAT5A | inhibition |
| PRKACA | ULK2 | inhibition |
| TARDBP | NDUFA1 | inhibition |
| PRKACB | OR51A2 | inhibition |
| PPP2R1B | CACNA2D2 | inhibition |
| PRKACB | MYLK | inhibition |
| MIR126 | PIK3CA | inhibition |
| PPP2R5C | AKT1 | inhibition |
| GRK3 | OR5H2 | inhibition |
| PPP1CC | GRIN2A | inhibition |
| PRKACA | OR6M1 | inhibition |
| SNCA | NDUFB10 | inhibition |
| NUMB | NOTCH3 | inhibition |
| GNG11 | CACNA1F | inhibition |
| PRKACB | OR2F2 | inhibition |
| GRK3 | OR52K1 | inhibition |
| WIF1 | WNT7A | inhibition |
| PIK3R3 | FOXO1 | inhibition |
| PPP1CB | CACNG2 | inhibition |
| BRAP | KSR2 | inhibition |
| IRAG1 | ITPR1 | inhibition |
| ARRB2 | OR2G3 | inhibition |
| PRKACG | OR5AU1 | inhibition |
| SOD1 | SDHD | inhibition |
| MAPT | NDUFA6 | inhibition |
| PLCB4 | KCNK3 | inhibition |
| PRKACG | OR10H1 | inhibition |
| ARRB1 | OR10G8 | inhibition |
| PRKACA | NDUFB11 | inhibition |
| PPP1CA | CAMK2B | inhibition |
| GNG11 | CACNA1D | inhibition |
| PRKACB | OR6C74 | inhibition |
| PPP2R2A | OCLN | inhibition |
| HTT | BECN1 | inhibition |
| PRKACA | NDUFB4 | inhibition |
| PRKACB | OR8B2 | inhibition |
| ARRB2 | OR4D2 | inhibition |
| SFRP1 | WNT10A | inhibition |
| CAMK2B | PDE4A | inhibition |
| ARRB1 | OR5T2 | inhibition |
| GRK2 | OR2AG2 | inhibition |
| PPP2R1A | CACNG8 | inhibition |
| CTSK | BIRC2 | inhibition |
| FNBP1L | CALCOCO2 | inhibition |
| MAP4K1 | MAP3K11 | phosphorylation |
| CAMK2G | FLNB | phosphorylation |
| MAPK11 | CDC25B | phosphorylation |
| GRK5 | CCR8 | phosphorylation |
| BUB1B-PAK6 | ARHGEF6 | phosphorylation |
| PAK1 | ARHGEF6 | phosphorylation |
| **GRK3** | CXCR6 | phosphorylation |
| MAPK13 | ELK4 | phosphorylation |
| ROCK1 | LIMK2 | phosphorylation |
| PAK2 | ARHGEF6 | phosphorylation |
| ROCK1 | PIP4K2B | phosphorylation |
| PAK2 | MAP3K3 | phosphorylation |
| MAPK11 | MAX | phosphorylation |
| MAPK11 | ELK4 | phosphorylation |
| MAPK1 | PLA2G4D | phosphorylation |
| IKBKB | NFKBIB | phosphorylation |
| MAPK3 | MYC | phosphorylation |
| GRK3 | CX3CR1 | phosphorylation |
| PAK1 | MAP3K3 | phosphorylation |
| MAPK11 | MAPKAPK3 | phosphorylation |
| PRKACA | RAP1B | phosphorylation |
| ROCK2 | PIP5K1A | phosphorylation |
| PRKCG | NRAS | phosphorylation |
| GRK5 | CCR5 | phosphorylation |
| CAMK2D | FLNB | phosphorylation |
| MAPKAPK5 | HSPB1 | phosphorylation |
| PAK5 | MAP2K1 | phosphorylation |
| GRK7 | CCR3 | phosphorylation |
| MAPK14 | MAPKAPK5 | phosphorylation |
| GRK5 | CCR7 | phosphorylation |
| ROCK1 | MYL7 | phosphorylation |
| RPS6KA2 | ATF4 | phosphorylation |
| MAPK13 | TP53 | phosphorylation |
| GRK1 | CCR9 | phosphorylation |
| MAPK1 | RPS6KA1 | phosphorylation |
| MAPK12 | NEFM | phosphorylation |
| MAP2K6 | MAPK13 | phosphorylation |
| GRK4 | CXCR4 | phosphorylation |
| MAPK12 | CDC25B | phosphorylation |
| PAK4 | ARHGEF7 | phosphorylation |
|  |  |  |

**Table S2**: Example prompts for identifying gene regulatory relationships.

| **Prompt1** | **Prompt3** |
| --- | --- |
| As a gene interaction analyst, your task is to determine the effect of {gene1} on {gene2} within the context of the KEGG Pathway Database, which represents our knowledge of molecular interaction, reaction, and relation networks for various biological processes. Provide a concise and definitive answer using one of the following terms: activation (gene1 activates gene2), inhibition (gene1 inhibits gene2), phosphorylation (gene1 phosphorylates gene2), or no information (no known relationship). Make sure your answer is definitive, composed of 'activation', 'inhibition', 'phosphorylation' or 'no information without further details or explanation.  Q: What effect does gene CCR5 have on gene GNB3?  Q: What effect does gene LIMK1 have on gene CASP1?  Q: What effect does gene IL17D have on gene IL2RB?  Q: What effect does gene OR4N2 have on gene GNG13?  Q: What effect does gene IL12A have on gene IL23R?  Q: What effect does gene CTSW have on gene XIAP?  Q: What effect does gene PRKACB have on gene OR8H3?  Q: What effect does gene GREM2 have on gene GDF6?  Q: What effect does gene LEFTY2 have on gene TGFB2?  Q: What effect does gene PTPN5 have on gene MAPK3?  Q: What effect does gene GRK3 have on gene CXCR6?  Q: What effect does gene ROCK1 have on gene LIMK2?  Q: What effect does gene MAP4K1 have on gene MAP3K11?  Q: What effect does gene CAMK2G have on gene FLNB?  Q: What effect does gene MAPK11 have on gene CDC25B? | As a gene interaction specialist, your task is to determine the effect of {gene1} on {gene2} using the KEGG Pathway Database, a comprehensive resource for understanding molecular interaction and relation networks in cellular processes. Provide a clear and unambiguous answer using one of the following terms: 'activation' (gene1 activates gene2), 'inhibition' (gene1 inhibits gene2), 'phosphorylation' (gene1 phosphorylates gene2), or 'no information' if there is no known relationship. Ensure your answer is definitive and based on the current knowledge in the KEGG Pathway Database, composed of 'activation', 'inhibition’,’ phosphorylation', or 'no information' without further details or explanation.  Q: What effect does gene CCR5 have on gene GNB3?  Q: What effect does gene LIMK1 have on gene CASP1?  Q: What effect does gene IL17D have on gene IL2RB?  Q: What effect does gene OR4N2 have on gene GNG13?  Q: What effect does gene IL12A have on gene IL23R?  Q: What effect does gene CTSW have on gene XIAP?  Q: What effect does gene PRKACB have on gene OR8H3?  Q: What effect does gene GREM2 have on gene GDF6?  Q: What effect does gene LEFTY2 have on gene TGFB2?  Q: What effect does gene PTPN5 have on gene MAPK3?  Q: What effect does gene GRK3 have on gene CXCR6?  Q: What effect does gene ROCK1 have on gene LIMK2?  Q: What effect does gene MAP4K1 have on gene MAP3K11?  Q: What effect does gene CAMK2G have on gene FLNB?  Q: What effect does gene MAPK11 have on gene CDC25B? |
| **Prompt3** | **Prompt4** |
| As a gene expression analyst, you are tasked with determining the effect of {gene1} on {gene2} using the KEGG Pathway Database, a valuable resource for understanding molecular interaction networks in biological processes. Your answer should only include one of the following terms: 'activation' (gene1 activates gene2), 'inhibition' (gene1 inhibits gene2), or 'phosphorylation' (gene1 phosphorylates gene2). Make sure your answer is definitive, composed of 'activation', 'inhibition', 'phosphorylation' or 'no information without further details or explanation.  Q: What effect does gene CCR5 have on gene GNB3?  Q: What effect does gene LIMK1 have on gene CASP1?  Q: What effect does gene IL17D have on gene IL2RB?  Q: What effect does gene OR4N2 have on gene GNG13?  Q: What effect does gene IL12A have on gene IL23R?  Q: What effect does gene CTSW have on gene XIAP?  Q: What effect does gene PRKACB have on gene OR8H3?  Q: What effect does gene GREM2 have on gene GDF6?  Q: What effect does gene LEFTY2 have on gene TGFB2?  Q: What effect does gene PTPN5 have on gene MAPK3?  Q: What effect does gene GRK3 have on gene CXCR6?  Q: What effect does gene ROCK1 have on gene LIMK2?  Q: What effect does gene MAP4K1 have on gene MAP3K11?  Q: What effect does gene CAMK2G have on gene FLNB?  Q: What effect does gene MAPK11 have on gene CDC25B? | As a genomics analyst, your task is to determine the effect of {gene1} on {gene2} using the KEGG Pathway Database, a comprehensive resource for understanding molecular interaction networks involved in cellular processes. Provide a clear and unambiguous answer using one of the following terms: 'activation' (gene1 activates gene2), 'inhibition' (gene1 inhibits gene2), or 'phosphorylation' (gene1 phosphorylates gene2). Ensure your answer is definitive and composed of 'activation', 'inhibition', 'phosphorylation', or 'no information without further details or explanation.  Q: What effect does gene CCR5 have on gene GNB3?  Q: What effect does gene LIMK1 have on gene CASP1?  Q: What effect does gene IL17D have on gene IL2RB?  Q: What effect does gene OR4N2 have on gene GNG13?  Q: What effect does gene IL12A have on gene IL23R?  Q: What effect does gene CTSW have on gene XIAP?  Q: What effect does gene PRKACB have on gene OR8H3?  Q: What effect does gene GREM2 have on gene GDF6?  Q: What effect does gene LEFTY2 have on gene TGFB2?  Q: What effect does gene PTPN5 have on gene MAPK3?  Q: What effect does gene GRK3 have on gene CXCR6?  Q: What effect does gene ROCK1 have on gene LIMK2?  Q: What effect does gene MAP4K1 have on gene MAP3K11?  Q: What effect does gene CAMK2G have on gene FLNB?  Q: What effect does gene MAPK11 have on gene CDC25B? |
| **Prompt5** | **Prompt6** |
| As a network biologist, your task is to determine the effect of {gene1} on {gene2} using the KEGG Pathway Database, a comprehensive resource for understanding molecular interaction networks in various biological processes. Provide a clear and unambiguous answer using one of the following terms: 'activation' (gene1 activates gene2), 'inhibition' (gene1 inhibits gene2), or 'phosphorylation' (gene1 phosphorylates gene2). Ensure your answer is based on the current knowledge in the KEGG Pathway Database and composed of 'activation', 'inhibition', or 'phosphorylation' without further details or explanation. Make sure your answer is definitive, composed of 'activation', 'inhibition', 'phosphorylation' or 'no information without further details or explanation.  Q: What effect does gene CCR5 have on gene GNB3?  Q: What effect does gene LIMK1 have on gene CASP1?  Q: What effect does gene IL17D have on gene IL2RB?  Q: What effect does gene OR4N2 have on gene GNG13?  Q: What effect does gene IL12A have on gene IL23R?  Q: What effect does gene CTSW have on gene XIAP?  Q: What effect does gene PRKACB have on gene OR8H3?  Q: What effect does gene GREM2 have on gene GDF6?  Q: What effect does gene LEFTY2 have on gene TGFB2?  Q: What effect does gene PTPN5 have on gene MAPK3?  Q: What effect does gene GRK3 have on gene CXCR6?  Q: What effect does gene ROCK1 have on gene LIMK2?  Q: What effect does gene MAP4K1 have on gene MAP3K11?  Q: What effect does gene CAMK2G have on gene FLNB?  Q: What effect does gene MAPK11 have on gene CDC25B? | As a system biologist, your task is to determine the effect of {gene1} on {gene2} using the KEGG Pathway Database, a comprehensive resource for understanding molecular interaction and relation networks in cellular processes. Provide a clear and unambiguous answer using one of the following terms: 'activation' (gene1 activates gene2), 'inhibition' (gene1 inhibits gene2), 'phosphorylation' (gene1 phosphorylates gene2), or 'no information' if there is no known relationship. Ensure your answer is definitive and composed of 'activation', 'inhibition', 'phosphorylation', or 'no information without further details or explanation.  Q: What effect does gene CCR5 have on gene GNB3?  Q: What effect does gene LIMK1 have on gene CASP1?  Q: What effect does gene IL17D have on gene IL2RB?  Q: What effect does gene OR4N2 have on gene GNG13?  Q: What effect does gene IL12A have on gene IL23R?  Q: What effect does gene CTSW have on gene XIAP?  Q: What effect does gene PRKACB have on gene OR8H3?  Q: What effect does gene GREM2 have on gene GDF6?  Q: What effect does gene LEFTY2 have on gene TGFB2?  Q: What effect does gene PTPN5 have on gene MAPK3?  Q: What effect does gene GRK3 have on gene CXCR6?  Q: What effect does gene ROCK1 have on gene LIMK2?  Q: What effect does gene MAP4K1 have on gene MAP3K11?  Q: What effect does gene CAMK2G have on gene FLNB?  Q: What effect does gene MAPK11 have on gene CDC25B? |
| **Prompt7 = Final prompt**  As a computational biologist, determine what is the relation between {gene1} and {gene2} in KEGG Pathway Database. KEGG pathways are collections of pathway maps that represent our knowledge of the molecular interaction, reaction, and relation networks for various biological processes. To determine the relation between {gene1} and {gene2}, please provide a definitive answer without further thought. Describe the relation in one or two of the following words: activation, inhibition, phosphorylation. If you are replying with two relations, separate two relations using '...'. Please note that the directionality of the relationship is important. Make sure your answer is definitive, answer with relations only, without further details or explanations. Make sure your answer is definitive, composed of 'activation', 'inhibition', 'phosphorylation' or 'no information without further details or explanation.  Q: What effect does gene CCR5 have on gene GNB3?  Q: What effect does gene LIMK1 have on gene CASP1?  Q: What effect does gene IL17D have on gene IL2RB?  Q: What effect does gene OR4N2 have on gene GNG13?  Q: What effect does gene IL12A have on gene IL23R?  Q: What effect does gene CTSW have on gene XIAP?  Q: What effect does gene PRKACB have on gene OR8H3?  Q: What effect does gene GREM2 have on gene GDF6?  Q: What effect does gene LEFTY2 have on gene TGFB2?  Q: What effect does gene PTPN5 have on gene MAPK3?  Q: What effect does gene GRK3 have on gene CXCR6?  Q: What effect does gene ROCK1 have on gene LIMK2?  Q: What effect does gene MAP4K1 have on gene MAP3K11?  Q: What effect does gene CAMK2G have on gene FLNB?  Q: What effect does gene MAPK11 have on gene CDC25B? | |

**Table S3**: Selected gene relationships for *ad hoc* testing

| **Number** | **Color** | **Starter** | **Receiver** | **Ground-truth Relation** |
| --- | --- | --- | --- | --- |
| **1-5** | **_____** | CCR5 | GNB3 | Activation |
|  |  | LIMK1 | CASP1 | Activation |
|  |  | IL17D | IL2RB | Activation |
|  |  | OR4N2 | GNG13 | Activation |
|  |  | IL12A | IL23R | Activation |
| **6 -10** | **_____** | CTSW | XIAP | Inhibition |
|  |  | PRKACB | OR8H3 | Inhibition |
|  |  | GREM2 | GDF6 | Inhibition |
|  |  | LEFTY2 | TGFB2 | Inhibition |
|  |  | PTPN5 | MAPK3 | Inhibition |
| **11-15** | _____ | GRK3 | CXCR6 | Phosphorylation |
|  |  | ROCK1 | LIMK2 | Phosphorylation |
|  |  | MAP4K1 | MAP3K11 | Phosphorylation |
|  |  | CAMK2G | FLNB | Phosphorylation |
|  |  | MAPK11 | CDC25B | Phosphorylation |

**Table S4**: Predicted gene regulatory relationships by the 21 LLMs using the seven prompts in Table S2 for the 15 gene relationships in Table S3

| **Model** | **Prompt1** | **Prompt2** | **Prompt3** | **Prompt4** | **Prompt5** | **Prompt6** | **Prompt7** |
| --- | --- | --- | --- | --- | --- | --- | --- |
| **Claude** | Activation  Activation  No knowledge  Inhibition  Inhibition  No knowledge  Inhibition  Inhibition  No knowledge  No knowledge  No knowledge  Inhibition  Inhibition  No knowledge  No knowledge | Activation  No knowledge  Activation  Activation  No knowledge  Activation  Activation  Inhibition  Inhibition  No knowledge  No knowledge  Activation  Activation  No knowledge  No knowledge | Activation  Activation  No knowledge  Activation  No knowledge  No knowledge  Inhibition  Inhibition  Activation  No knowledge  Phosphorylation  No knowledge  No knowledge  No knowledge  No knowledge | No knowledge  Activation  Activation  No knowledge  No knowledge  Inhibition  No knowledge  Inhibition  Inhibition  No knowledge  No knowledge  Phosphorylation  Inhibition  Inhibition  No knowledge | Activation  Activation  Inhibition  Inhibition  No knowledge  No knowledge  Inhibition  No knowledge  Activation  Activation  No knowledge  No knowledge  No knowledge  No knowledge  No knowledge | No knowledge  Activation  Activation  No knowledge  Inhibition  No knowledge  Inhibition  Inhibition  No knowledge  No knowledge  No knowledge  No knowledge  No knowledge  No knowledge  No knowledge | Activation  Activation  No knowledge  Activation  No knowledge  No knowledge  Inhibition  Inhibition  No knowledge  No knowledge  No knowledge  Phosphorylation  Phosphorylation  Activation  Activation |
| **Claude-Instant** | Inhibition  Inhibition  Activation  Activation  No knowledge  No knowledge  No knowledge  No knowledge  Inhibition  Inhibition  No knowledge  No knowledge  No knowledge  No knowledge  No knowledge | Activation  No knowledge  Activation  Inhibition  Inhibition  No knowledge  No knowledge  Inhibition  Inhibition  Inhibition  Activation  Activation  No knowledge  Phosphorylation  Phosphorylation | Activation  No knowledge  Activation  Activation  No knowledge  No knowledge  No knowledge  Inhibition  Inhibition  Inhibition  No knowledge  No knowledge  No knowledge  Phosphorylation  Phosphorylation | No knowledge  Activation  Activation  No knowledge  Inhibition  No knowledge  Inhibition  Inhibition  Inhibition  No knowledge  No knowledge  Phosphorylation  No knowledge  Activation  Activation | Activation  Activation  Inhibition  No knowledge  No knowledge  No knowledge  Inhibition  No knowledge  No knowledge  No knowledge  Inhibition  No knowledge  Phosphorylation  No knowledge  No knowledge | Activation  No knowledge  Activation  Activation  No knowledge  No knowledge  No knowledge  Inhibition  Inhibition  Inhibition  Inhibition  Inhibition  No knowledge  Phosphorylation  Phosphorylation | Activation  Activation  No knowledge  Activation  No knowledge  No knowledge  Inhibition  Inhibition  No knowledge  Inhibition  Activation  Activation  Phosphorylation  No knowledge  Phosphorylation |
| **cohere** | Activation  No knowledge  Activation  Inhibition  Inhibition  No knowledge  Inhibition  No knowledge  Inhibition  No knowledge  No knowledge  Inhibition  Activation  Activation  Activation | Activation  No knowledge  Activation  Activation  No knowledge  No knowledge  No knowledge  Inhibition  Inhibition  No knowledge  No knowledge  Inhibition  Inhibition  Inhibition  No knowledge | Activation  Activation  No knowledge  No knowledge  No knowledge  Inhibition  Inhibition  Inhibition  No knowledge  No knowledge  No knowledge  Phosphorylation  Inhibition  Inhibition  No knowledge | No knowledge  Activation  Activation  No knowledge  No knowledge  Activation  No knowledge  Inhibition  Inhibition  No knowledge  Phosphorylation  Phosphorylation  No knowledge  No knowledge  No knowledge | Activation  Activation  No knowledge  Activation  No knowledge  No knowledge  Inhibition  No knowledge  Inhibition  No knowledge  No knowledge  No knowledge  Phosphorylation  Activation  Activation | No knowledge  Activation  Activation  No knowledge  No knowledge  No knowledge  Inhibition  Inhibition  No knowledge  No knowledge  Phosphorylation  No knowledge  Inhibition  Inhibition  No knowledge | Activation  Activation  No knowledge  Activation  No knowledge  No knowledge  Inhibition  Inhibition  No knowledge  Inhibition  No knowledge  Phosphorylation  Phosphorylation  No knowledge  Phosphorylation |
| **GPT-3.5** | Activation  Activation  Inhibition  Inhibition  No knowledge  No knowledge  Inhibition  Inhibition  No knowledge  No knowledge  Phosphorylation  Phosphorylation  No knowledge  No knowledge  No knowledge | Activation  No knowledge  Activation  Activation  No knowledge  No knowledge  No knowledge  Inhibition  Inhibition  No knowledge  Inhibition  Inhibition  Inhibition  Inhibition  No knowledge | No knowledge  Activation  No knowledge  Activation  No knowledge  No knowledge  Inhibition  Inhibition  No knowledge  Inhibition  Phosphorylation  Activation  Activation  Activation  Activation | No knowledge  Activation  Activation  No knowledge  No knowledge  Activation  No knowledge  Inhibition  No knowledge  No knowledge  No knowledge  Phosphorylation  No knowledge  No knowledge  Phosphorylation | Activation  Activation  Inhibition  Inhibition  No knowledge  No knowledge  Inhibition  No knowledge  No knowledge  No knowledge  No knowledge  No knowledge  Phosphorylation  No knowledge  No knowledge | No knowledge  Activation  Activation  No knowledge  No knowledge  No knowledge  Inhibition  Inhibition  No knowledge  No knowledge  Activation  Activation  Phosphorylation  No knowledge  No knowledge | Activation  Activation  No knowledge  Activation  No knowledge  No knowledge  Inhibition  Inhibition  No knowledge  Inhibition  No knowledge  Phosphorylation  Phosphorylation  No knowledge  No knowledge |
| **GPT-4** | Activation  Activation  No knowledge  Activation  No knowledge  No knowledge  Inhibition  Inhibition  No knowledge  Inhibition  Inhibition  Inhibition  Phosphorylation  No knowledge  No knowledge | No knowledge  Activation  Activation  Activation  No knowledge  No knowledge  Inhibition  No knowledge  No knowledge  Inhibition  No knowledge  No knowledge  No knowledge No knowledge  No knowledge | Activation  Activation  No knowledge  Activation  No knowledge  No knowledge  Inhibition  Inhibition  No knowledge  No knowledge  Phosphorylation  Phosphorylation  No knowledge  No knowledge  No knowledge | No knowledge  Activation  Activation  No knowledge  Activation  No knowledge  No knowledge  Inhibition  Inhibition  No knowledge  No knowledge  Phosphorylation  No knowledge  No knowledge  No knowledge | Activation  Activation  No knowledge  No knowledge  Inhibition  No knowledge  Inhibition  No knowledge  No knowledge  No knowledge  No knowledge  No knowledge  Phosphorylation  No knowledge  No knowledge | No knowledge  Activation  Activation  No knowledge  Activation  No knowledge  Inhibition  Inhibition  No knowledge  No knowledge  No knowledge  No knowledge  Phosphorylation  Phosphorylation  No knowledge | Activation  Activation  No knowledge  Activation  Activation  No knowledge  Inhibition  Inhibition  No knowledge  Inhibition  No knowledge  Phosphorylation  Phosphorylation  No knowledge  Phosphorylation |
| **Claude-Pro** | No knowledge  Activation  Activation  No knowledge  No knowledge  No knowledge  Inhibition  Inhibition  No knowledge  Inhibition  No knowledge  No knowledge  Phosphorylation  No knowledge  No knowledge | No knowledge  Activation  Activation  No knowledge  No knowledge  No knowledge  Inhibition  No knowledge  No knowledge  Inhibition  No knowledge  No knowledge  Phosphorylation  No knowledge  No knowledge | Activation  Activation  No knowledge  Activation  No knowledge  No knowledge  Inhibition  Inhibition  No knowledge  No knowledge  Phosphorylation  No knowledge  No knowledge Phosphorylation  No knowledge | Activation  Activation  No knowledge  No knowledge  Activation  No knowledge  No knowledge  Inhibition  Inhibition  No knowledge  No knowledge  Phosphorylation  No knowledge  No knowledge  Phosphorylation | Activation  Activation  No knowledge  No knowledge  Activation  Inhibition  No knowledge  Inhibition  No knowledge  Inhibition  No knowledge  No knowledge  No knowledge  Phosphorylation  No knowledge | No knowledge  Activation  Activation  No knowledge  Activation  No knowledge  Inhibition  Inhibition  No knowledge  No knowledge  Inhibition  No knowledge  Phosphorylation  Phosphorylation  No knowledge | Activation  Activation  Activation  Activation  Inhibition  No knowledge  Inhibition  Inhibition  No knowledge  Inhibition  No knowledge  Phosphorylation  Phosphorylation  Phosphorylation  No knowledge |
| **Palm2** | Activation  No knowledge  Activation  Activation  No knowledge  No knowledge  Activation  Activation  Inhibition  No knowledge  Inhibition  No knowledge  Activation  Activation  Activation | Activation  No knowledge  Activation  Activation  No knowledge  No knowledge  No knowledge  Inhibition  Inhibition  Inhibition  No knowledge  No knowledge  No knowledge  Phosphorylation  Phosphorylation | Activation  No knowledge  Activation  Activation  No knowledge  No knowledge  No knowledge  Activation  Inhibition  Inhibition  No knowledge  No knowledge  No knowledge  Phosphorylation  Phosphorylation | No knowledge  Activation  Activation  Activation  No knowledge  No knowledge  Activation  Inhibition  Inhibition  No knowledge  No knowledge  Phosphorylation  No knowledge  No knowledge  No knowledge | Activation  Activation  Inhibition  Inhibition  No knowledge  No knowledge  Inhibition  Activation  Activation  No knowledge  Inhibition  Inhibition  Phosphorylation  No knowledge  No knowledge | Activation  No knowledge  Activation  Activation  No knowledge  No knowledge  No knowledge  Inhibition  Inhibition  Inhibition  No knowledge  No knowledge  No knowledge  Phosphorylation  Phosphorylation | Activation  Activation  No knowledge  Activation  No knowledge  No knowledge  Inhibition  Inhibition  No knowledge  Inhibition  No knowledge  No knowledge  Phosphorylation  No knowledge  Phosphorylation |
| **Bard** | Activation  Activation  No knowledge  No knowledge  No knowledge  No knowledge  Inhibition  Inhibition  No knowledge  No knowledge  No knowledge  No knowledge  No knowledge  No knowledge  No knowledge | Activation  No knowledge  Activation  Activation  No knowledge  No knowledge  No knowledge  Inhibition  Inhibition  No knowledge  No knowledge  No knowledge  No knowledge  No knowledge  No knowledge | Activation  Activation  No knowledge  Activation  No knowledge  No knowledge  Inhibition  Inhibition  No knowledge  No knowledge  Phosphorylation  No knowledge  No knowledge  No knowledge  No knowledge | No knowledge  Activation  Activation  No knowledge  No knowledge  No knowledge  No knowledge  Inhibition  Inhibition  No knowledge  No knowledge  Phosphorylation  No knowledge  No knowledge  No knowledge | Activation  Activation  No knowledge  No knowledge  No knowledge  No knowledge  Inhibition  No knowledge  No knowledge  No knowledge  No knowledge  No knowledge  Phosphorylation  No knowledge  No knowledge | No knowledge  Activation  Activation  No knowledge  No knowledge  No knowledge  Inhibition  Inhibition  No knowledge  No knowledge  No knowledge  No knowledge  No knowledge  No knowledge  No knowledge | Activation  Activation  No knowledge  Activation  No knowledge  No knowledge  Inhibition  Inhibition  No knowledge  No knowledge  No knowledge  Phosphorylation  Phosphorylation  No knowledge  No knowledge |
| **Codellama-34** | No knowledge  Activation  No knowledge  No knowledge  No knowledge  No knowledge  Inhibition  No knowledge  Inhibition  No knowledge  Phosphorylation  No knowledge  No knowledge  Phosphorylation  No knowledge | Activation  No knowledge  Activation  Activation  No knowledge  No knowledge  No knowledge  Inhibition  No knowledge  Inhibition  No knowledge  No knowledge  No knowledge  No knowledge  No knowledge | No knowledge  Activation  No knowledge  Activation  No knowledge  No knowledge  Inhibition  Inhibition  No knowledge  Inhibition  Phosphorylation  No knowledge  No knowledge  No knowledge  No knowledge | No knowledge  Activation  Activation  No knowledge  No knowledge  Activation  No knowledge  Inhibition  No knowledge  No knowledge  No knowledge  Phosphorylation  No knowledge  No knowledge  Phosphorylation | Activation  Activation  No knowledge  No knowledge  No knowledge  No knowledge  Inhibition  No knowledge  No knowledge  No knowledge  No knowledge  No knowledge  Phosphorylation  No knowledge  No knowledge | No knowledge  Activation  Activation  No knowledge  No knowledge  No knowledge  Inhibition  Inhibition  No knowledge  No knowledge  No knowledge  No knowledge  Phosphorylation  No knowledge  No knowledge | Activation  Activation  No knowledge  Activation  No knowledge  No knowledge  Inhibition  Inhibition  No knowledge  Inhibition  No knowledge  Phosphorylation  Phosphorylation  No knowledge  No knowledge |
| **Wizardlm-70** | No knowledge  No knowledge  Activation  Activation  No knowledge  No knowledge  Inhibition  Inhibition  No knowledge  No knowledge  Phosphorylation  Phosphorylation  No knowledge  No knowledge  No knowledge | No knowledge  Activation  Activation  Activation  No knowledge  No knowledge  No knowledge  Inhibition  Inhibition  No knowledge  No knowledge  No knowledge  No knowledge  No knowledge  No knowledge | Activation  No knowledge  No knowledge  Activation  No knowledge  No knowledge  Inhibition  Inhibition  No knowledge  Inhibition  Phosphorylation  No knowledge  No knowledge  No knowledge  No knowledge | Activation  Activation  No knowledge  No knowledge  No knowledge  Activation  No knowledge  Inhibition  No knowledge  No knowledge  No knowledge  Phosphorylation  No knowledge  No knowledge  Phosphorylation | Activation  Activation  No knowledge  No knowledge  No knowledge  No knowledge  Inhibition  No knowledge  No knowledge  No knowledge  No knowledge  No knowledge  Phosphorylation  No knowledge  No knowledge | Activation  Activation  No knowledge  No knowledge  No knowledge  No knowledge  Inhibition  Inhibition  No knowledge  No knowledge  No knowledge  No knowledge  Phosphorylation  No knowledge  No knowledge | Activation  Activation  No knowledge  Activation  No knowledge  No knowledge  Inhibition  Inhibition  No knowledge  Inhibition  No knowledge  Phosphorylation  No knowledge  No knowledge  Phosphorylation |
| **Wizardlm-13** | No knowledge  Activation  No knowledge  Activation  No knowledge  No knowledge  Inhibition  No knowledge  No knowledge  Inhibition  No knowledge  No knowledge  No knowledge  Phosphorylation  No knowledge | Activation  No knowledge  Activation  No knowledge  Activation  No knowledge  No knowledge  Inhibition  Inhibition  No knowledge  No knowledge  No knowledge  No knowledge  No knowledge  No knowledge | No knowledge  Activation  Activation  No knowledge  Activation  No knowledge  No knowledge  Inhibition  Inhibition  No knowledge  No knowledge  Phosphorylation  No knowledge  No knowledge  No knowledge | No knowledge  Activation  Activation  No knowledge  No knowledge  No knowledge  No knowledge  Inhibition  Inhibition  No knowledge  No knowledge  Phosphorylation  No knowledge  No knowledge  No knowledge | Activation  Activation  No knowledge  No knowledge  No knowledge  No knowledge  Inhibition  No knowledge  No knowledge  No knowledge  No knowledge  No knowledge  Phosphorylation  No knowledge  No knowledge | No knowledge  Activation  Activation  No knowledge  No knowledge  No knowledge  Inhibition  Inhibition  No knowledge  No knowledge  No knowledge  No knowledge  No knowledge  No knowledge  No knowledge | Activation  Activation  No knowledge  Activation  No knowledge  No knowledge  Inhibition  Inhibition  No knowledge  No knowledge  Phosphorylation  Phosphorylation  No knowledge  No knowledge  No knowledge |
| **Falcon-180** | No knowledge  Activation  Activation  No knowledge  No knowledge  No knowledge  Inhibition  Inhibition  No knowledge  Inhibition  No knowledge  No knowledge  Phosphorylation  No knowledge  No knowledge | No knowledge  Activation  Activation  No knowledge  No knowledge  No knowledge  Inhibition  No knowledge  No knowledge  Inhibition  No knowledge  No knowledge  Phosphorylation  No knowledge  No knowledge | Activation  Activation  No knowledge  Activation  No knowledge  No knowledge  Inhibition  Inhibition  No knowledge  No knowledge  Phosphorylation  No knowledge  No knowledge Phosphorylation  No knowledge | Activation  Activation  No knowledge  No knowledge  Activation  No knowledge  No knowledge  Inhibition  Inhibition  No knowledge  No knowledge  Phosphorylation  No knowledge  No knowledge  Phosphorylation | Activation  Activation  No knowledge  No knowledge  Activation  Inhibition  No knowledge  Inhibition  No knowledge  Inhibition  Inhibition  No knowledge  No knowledge  No knowledge  Phosphorylation | No knowledge  Activation  Activation  No knowledge  Activation  No knowledge  Inhibition  Inhibition  No knowledge  No knowledge  Inhibition  No knowledge  Phosphorylation  Phosphorylation  No knowledge | No knowledge  Activation  Activation  No knowledge  Activation  No knowledge  Inhibition  Inhibition  No knowledge  Inhibition  No knowledge  Phosphorylation  Phosphorylation  No knowledge  No knowledge |
| **Mistral-7** | No knowledge  Activation  Activation  No knowledge  No knowledge  No knowledge  Inhibition  No knowledge  Inhibition  No knowledge  No knowledge  No knowledge  No knowledge  Phosphorylation  No knowledge | Activation  No knowledge  Activation  Activation  No knowledge  No knowledge  No knowledge  Inhibition  Inhibition  No knowledge  No knowledge  No knowledge  No knowledge  No knowledge  No knowledge | Activation  Activation  No knowledge  Activation  No knowledge  No knowledge  Inhibition  Inhibition  No knowledge  No knowledge  Phosphorylation  No knowledge  No knowledge  No knowledge  No knowledge | No knowledge  Activation  Activation  No knowledge  No knowledge  No knowledge  No knowledge  Inhibition  Inhibition  No knowledge  No knowledge  Phosphorylation  No knowledge  No knowledge  No knowledge | Activation  Activation  No knowledge  No knowledge  No knowledge  No knowledge  Inhibition  No knowledge  No knowledge  No knowledge  No knowledge  No knowledge  Phosphorylation  No knowledge  No knowledge | No knowledge  Activation  Activation  No knowledge  No knowledge  No knowledge  Inhibition  Inhibition  No knowledge  No knowledge  No knowledge  No knowledge  No knowledge  No knowledge  No knowledge | Activation  Activation  No knowledge  Activation  No knowledge  No knowledge  Inhibition  Inhibition  No knowledge  No knowledge  No knowledge  Phosphorylation  Phosphorylation  No knowledge  No knowledge |
| **Chatglm2-6** | Activation  No knowledge  No knowledge  No knowledge  No knowledge  No knowledge  Inhibition  Inhibition  No knowledge  No knowledge  Phosphorylation  No knowledge  Phosphorylation  No knowledge  No knowledge | Activation  No knowledge  Activation  Activation  No knowledge  No knowledge  No knowledge  Inhibition  Inhibition  No knowledge  No knowledge  No knowledge  No knowledge  No knowledge  No knowledge | No knowledge  Activation  No knowledge  Activation  No knowledge  No knowledge  Inhibition  Inhibition  No knowledge  Inhibition  Phosphorylation  No knowledge  Activation  Activation  No knowledge | Activation  Activation  No knowledge  No knowledge  No knowledge  Activation  No knowledge  Inhibition  No knowledge  No knowledge  No knowledge  Phosphorylation  No knowledge  No knowledge  Phosphorylation | No knowledge  No knowledge  Activation  Activation  No knowledge  No knowledge  Inhibition  No knowledge  No knowledge  No knowledge  No knowledge  No knowledge  Phosphorylation  Activation  Activation | Activation  No knowledge  Activation  No knowledge  No knowledge  No knowledge  Inhibition  No knowledge  Inhibition  No knowledge  No knowledge  No knowledge  Phosphorylation  No knowledge  No knowledge | Activation  Activation  No knowledge  Activation  No knowledge  No knowledge  Inhibition  Inhibition  No knowledge  Inhibition  No knowledge  Phosphorylation  Phosphorylation  Activation  Activation |
| **Vicuna-7b** | No knowledge  Activation  Activation  No knowledge  No knowledge  No knowledge  Inhibition  Inhibition  No knowledge  No knowledge  No knowledge  No knowledge  No knowledge  No knowledge  No knowledge | Activation  No knowledge  Activation  Activation  No knowledge  No knowledge  No knowledge  Inhibition  No knowledge  Inhibition  No knowledge  No knowledge  No knowledge  No knowledge  No knowledge | Activation  Activation  No knowledge  Activation  No knowledge  No knowledge  Inhibition  Inhibition  No knowledge  No knowledge  Phosphorylation  No knowledge  No knowledge  No knowledge  No knowledge | No knowledge  Activation  Activation  No knowledge  No knowledge  No knowledge  No knowledge  Inhibition  Inhibition  No knowledge  No knowledge  Phosphorylation  No knowledge  No knowledge  No knowledge | Activation  Activation  No knowledge  No knowledge  No knowledge  No knowledge  Inhibition  No knowledge  No knowledge  No knowledge  No knowledge  No knowledge  Phosphorylation  Activation  Activation | No knowledge  Activation  Activation  No knowledge  No knowledge  No knowledge  Inhibition  Inhibition  No knowledge  No knowledge  No knowledge  Activation  Activation  Activation  Activation | Activation  No knowledge  Activation  Activation  No knowledge  No knowledge  Inhibition  Inhibition  No knowledge  Inhibition  No knowledge  Phosphorylation  Phosphorylation  No knowledge  No knowledge |
| **Vicuna-33** | No knowledge  Activation  No knowledge  Activation  No knowledge  No knowledge  Inhibition  No knowledge  Inhibition  No knowledge  Phosphorylation  No knowledge  No knowledge  No knowledge  No knowledge | Activation  No knowledge  Activation  Activation  No knowledge  No knowledge  No knowledge  Inhibition  No knowledge  No knowledge  Inhibition  Inhibition  No knowledge  No knowledge  No knowledge | No knowledge  Activation  No knowledge  Activation  No knowledge  No knowledge  Inhibition  Inhibition  No knowledge  Inhibition  Phosphorylation  No knowledge  Inhibition  Inhibition  No knowledge | No knowledge  Activation  Activation  No knowledge  No knowledge  Activation  No knowledge  Inhibition  No knowledge  No knowledge  No knowledge  Phosphorylation  No knowledge  No knowledge  Phosphorylation | Activation  Activation  No knowledge  No knowledge  No knowledge  No knowledge  Inhibition  No knowledge  No knowledge  No knowledge  No knowledge  No knowledge  Phosphorylation  No knowledge  No knowledge | Activation  No knowledge  Activation  No knowledge  No knowledge  No knowledge  Inhibition  Inhibition  No knowledge  No knowledge  No knowledge  No knowledge  Inhibition  Inhibition  No knowledge | Activation  Activation  No knowledge  Activation  No knowledge  No knowledge  Inhibition  Inhibition  No knowledge  Inhibition  No knowledge  Phosphorylation  No knowledge  No knowledge  Phosphorylation |
| **Vicuna-13b** | Activation  Activation  No knowledge  No knowledge  No knowledge  No knowledge  Inhibition  Inhibition  No knowledge  No knowledge  No knowledge  Activation  Activation  No knowledge  No knowledge | No knowledge  Activation  Activation  Activation  No knowledge  No knowledge  No knowledge  Inhibition  Inhibition  Activation  Activation  No knowledge  No knowledge  No knowledge  No knowledge | Activation  Activation  No knowledge  Activation  No knowledge  No knowledge  Inhibition  Inhibition  No knowledge  No knowledge  Phosphorylation  Activation  Activation  No knowledge  No knowledge | No knowledge  Activation  Activation  No knowledge  No knowledge  No knowledge  No knowledge  Inhibition  Inhibition  No knowledge  No knowledge  Phosphorylation  Activation  Activation  No knowledge | Activation  Activation  No knowledge  Inhibition  Inhibition  No knowledge  Inhibition  No knowledge  No knowledge  Activation  Activation  No knowledge  Phosphorylation  No knowledge  No knowledge | No knowledge  Activation  Activation  No knowledge  No knowledge  No knowledge  Inhibition  Inhibition  No knowledge  Activation  Activation  No knowledge  No knowledge  Activation  Activation | No knowledge  Activation  Activation  Activation  No knowledge  No knowledge  Inhibition  Inhibition  Activation  Activation  No knowledge  Phosphorylation  Phosphorylation  Activation  Activation |
| **llama2-70b** | No knowledge  No knowledge  Activation  Activation  No knowledge  No knowledge  Inhibition  Inhibition  No knowledge  No knowledge  No knowledge  Inhibition  Inhibition  Inhibition  Inhibition | Activation  No knowledge  Activation  Activation  No knowledge  No knowledge  No knowledge  Inhibition  Inhibition  No knowledge  No knowledge  Inhibition  Inhibition  Activation  Activation | No knowledge  Activation  Activation  Activation  No knowledge  No knowledge  Inhibition  No knowledge  Inhibition  No knowledge  Phosphorylation  No knowledge  No knowledge  Inhibition  Activation | No knowledge  Activation  Activation  No knowledge  No knowledge  No knowledge  No knowledge  Inhibition  Inhibition  No knowledge  No knowledge  Phosphorylation  No knowledge  No knowledge  No knowledge | No knowledge  No knowledge  Activation  Activation  No knowledge  No knowledge  Inhibition  No knowledge  No knowledge  No knowledge  No knowledge  No knowledge  Phosphorylation  No knowledge  No knowledge | Activation  Activation  No knowledge  No knowledge  No knowledge  No knowledge  Inhibition  Inhibition  No knowledge  No knowledge  No knowledge  Inhibition  Activation  No knowledge  No knowledge | Activation  Activation  No knowledge  No knowledge  Activation  Inhibition  No knowledge  Inhibition  Activation  Activation  Phosphorylation  No knowledge  Phosphorylation  No knowledge  No knowledge |
| **llama2-13b** | Activation  Activation  No knowledge  No knowledge  No knowledge  No knowledge  Inhibition  Inhibition  No knowledge  No knowledge  Activation  Activation  No knowledge  No knowledge  No knowledge | Activation  No knowledge  Activation  Activation  No knowledge  No knowledge  No knowledge  Inhibition  Inhibition  No knowledge  No knowledge  No knowledge  Activation  Activation  No knowledge | Activation  Activation  No knowledge  Activation  No knowledge  No knowledge  Inhibition  Inhibition  No knowledge  No knowledge  Phosphorylation  Activation  Activation  No knowledge  No knowledge | No knowledge  Activation  Activation  No knowledge  No knowledge  No knowledge  No knowledge  Inhibition  Inhibition  No knowledge  No knowledge  Phosphorylation  Activation  Activation  No knowledge | Activation  Activation  No knowledge  No knowledge  No knowledge  No knowledge  Inhibition  No knowledge  No knowledge  No knowledge  No knowledge  No knowledge  Phosphorylation  Activation  Activation | No knowledge  Activation  Activation  No knowledge  No knowledge  No knowledge  Inhibition  Inhibition  No knowledge  No knowledge  No knowledge  Inhibition  Inhibition  No knowledge  No knowledge | No knowledge  Activation  Activation  Activation  No knowledge  No knowledge  Inhibition  Inhibition  No knowledge  No knowledge  No knowledge  Phosphorylation  Phosphorylation  No knowledge  No knowledge |
| **llama2-7b** | No knowledge  No knowledge  Activation  Activation  No knowledge  No knowledge  Inhibition  Inhibition  No knowledge  No knowledge  Phosphorylation  No knowledge  Phosphorylation  No knowledge  No knowledge | Activation  No knowledge  Activation  Activation  No knowledge  No knowledge  No knowledge  Inhibition  No knowledge  Inhibition  No knowledge  No knowledge  No knowledge  No knowledge  No knowledge | No knowledge  Activation  No knowledge  Activation  No knowledge  No knowledge  Inhibition  Inhibition  No knowledge  Inhibition  Phosphorylation  Activation  Activation  No knowledge  No knowledge | No knowledge  Activation  Activation  No knowledge  No knowledge  Activation  No knowledge  Inhibition  No knowledge  No knowledge  No knowledge  Phosphorylation  Activation  Activation  Phosphorylation | Activation  Activation  No knowledge  No knowledge  No knowledge  No knowledge  Inhibition  No knowledge  No knowledge  No knowledge  No knowledge  No knowledge  Phosphorylation  Activation  Activation | No knowledge  Activation  Activation  No knowledge  No knowledge  No knowledge  Inhibition  Inhibition  No knowledge  No knowledge  No knowledge  No knowledge  Phosphorylation  No knowledge  No knowledge | Activation  Activation  No knowledge  Activation  No knowledge  No knowledge  Inhibition  Inhibition  No knowledge  Inhibition  No knowledge  Phosphorylation  Activation  Activation  Phosphorylation |
| **Qwen-14b** | Activation  No knowledge  No knowledge  No knowledge  No knowledge  No knowledge  No knowledge  No knowledge  No knowledge  No knowledge  Phosphorylation  Phosphorylation  No knowledge  No knowledge  No knowledge | No knowledge  Activation  No knowledge  Activation  Activation  No knowledge  No knowledge  No knowledge  Inhibition  No knowledge  Inhibition  No knowledge  No knowledge  No knowledge  No knowledge | Activation  No knowledge  Activation  No knowledge  No knowledge  No knowledge  No knowledge  Inhibition  Inhibition  No knowledge  Inhibition  No knowledge  No knowledge  No knowledge  No knowledge | No knowledge  Activation  No knowledge  Activation  No knowledge  No knowledge  Activation  No knowledge  Inhibition  No knowledge  No knowledge  Phosphorylation  No knowledge  No knowledge  No knowledge | No knowledge  No knowledge  Activation  Activation  No knowledge  No knowledge  Inhibition  No knowledge  No knowledge  No knowledge  No knowledge  No knowledge  Phosphorylation  No knowledge  No knowledge | Activation  No knowledge  Activation  No knowledge  No knowledge  No knowledge  Inhibition  Inhibition  No knowledge  No knowledge  No knowledge  No knowledge  Phosphorylation  No knowledge  No knowledge | No knowledge  Activation  Activation  No knowledge  Activation  No knowledge  No knowledge  Inhibition  Inhibition  No knowledge  Inhibition  No knowledge  Phosphorylation  No knowledge  No knowledge |

**Table S5**: Predicted gene regulatory relationships by the 21 LLMs with varying temperatures for the 15 gene relationships in Table S3

| **Model** | **Temperature =0.2** | **Temperature =0.4** | **Temperature =0.6** | **Temperature =0.8** | **Temperature =0.9** |
| --- | --- | --- | --- | --- | --- |
| **cohere** | No knowledge  No knowledge  No knowledge  No knowledge  No knowledge  No knowledge  No knowledge  No knowledge  No knowledge  No knowledge  No knowledge  No knowledge  No knowledge  No knowledge  No knowledge | No knowledge  No knowledge  No knowledge  No knowledge  No knowledge  No knowledge  No knowledge  No knowledge  No knowledge  No knowledge  No knowledge  No knowledge  No knowledge  No knowledge  No knowledge | No knowledge  No knowledge  No knowledge  No knowledge  No knowledge  No knowledge  No knowledge  No knowledge  No knowledge  No knowledge  No knowledge  No knowledge  No knowledge  No knowledge  No knowledge | No knowledge  Inhibition  Activation  Activation  No knowledge  No knowledge  Activation  Inhibition  Inhibition  No knowledge  Activation  Activation  No knowledge  No knowledge  Phosphorylation | Activation  No knowledge  Activation  Activation  No knowledge  No knowledge  Activation  Inhibition  Inhibition  No knowledge  No knowledge  Activation  Activation  No knowledge  Phosphorylation |
| **GPT-3.5** | No knowledge  No knowledge  No knowledge  No knowledge  No knowledge  No knowledge  No knowledge  No knowledge  No knowledge  No knowledge  No knowledge  No knowledge  No knowledge  No knowledge  No knowledge | No knowledge  No knowledge  No knowledge  No knowledge  No knowledge  No knowledge  No knowledge  No knowledge  No knowledge  No knowledge  No knowledge  No knowledge  No knowledge  No knowledge  No knowledge | No knowledge  No knowledge  No knowledge  No knowledge  No knowledge  No knowledge  No knowledge  No knowledge  No knowledge  No knowledge  No knowledge  No knowledge  No knowledge  No knowledge  No knowledge | Activation  Activation  No knowledge  Inhibition  No knowledge  No knowledge  No knowledge  Inhibition  No knowledge  Activation  No knowledge  Activation  No knowledge  No knowledge  Phosphorylation | Activation  No knowledge  Activation  Inhibition  Activation  No knowledge  Activation  Inhibition  Inhibition  No knowledge  Activation  No knowledge  No knowledge  Inhibition  Phosphorylation |
| **GPT-4** | No knowledge  No knowledge  No knowledge  No knowledge  No knowledge  No knowledge  No knowledge  No knowledge  No knowledge  No knowledge  No knowledge  No knowledge  No knowledge  No knowledge  No knowledge | No knowledge  No knowledge  No knowledge  No knowledge  No knowledge  No knowledge  No knowledge  No knowledge  No knowledge  No knowledge  No knowledge  No knowledge  No knowledge  No knowledge  No knowledge | No knowledge  No knowledge  No knowledge  No knowledge  No knowledge  No knowledge  No knowledge  No knowledge  No knowledge  No knowledge  No knowledge  No knowledge  No knowledge  No knowledge  No knowledge | No knowledge  Activation  Inhibition  Activation  Activation  Inhibition  No knowledge  No knowledge  Inhibition  Inhibition  No knowledge  Activation  Activation  Phosphorylation  Phosphorylation | Activation  Activation  Activation  Activation  Inhibition  No knowledge  No knowledge  Inhibition  Inhibition  Inhibition  No knowledge  Activation  Phosphorylation  Phosphorylation  Phosphorylation |
| **Claude-Pro** | No knowledge  No knowledge  No knowledge  No knowledge  No knowledge  No knowledge  No knowledge  No knowledge  No knowledge  No knowledge  No knowledge  No knowledge  No knowledge  No knowledge  No knowledge | No knowledge  No knowledge  No knowledge  No knowledge  No knowledge  No knowledge  No knowledge  No knowledge  No knowledge  No knowledge  No knowledge  No knowledge  No knowledge  No knowledge  No knowledge | No knowledge  No knowledge  No knowledge  No knowledge  No knowledge  No knowledge  No knowledge  No knowledge  No knowledge  No knowledge  No knowledge  No knowledge  No knowledge  No knowledge  No knowledge | No knowledge  No knowledge  No knowledge  No knowledge  No knowledge  No knowledge  No knowledge  No knowledge  No knowledge  No knowledge  No knowledge  No knowledge  No knowledge  No knowledge  No knowledge | Activation  Activation  Activation  Activation  No knowledge  No knowledge  Inhibition  Activation  Inhibition  Inhibition  No knowledge  Inhibition  No knowledge  Phosphorylation  Phosphorylation |
| **Claude** | No knowledge  No knowledge  No knowledge  No knowledge  No knowledge  No knowledge  No knowledge  No knowledge  No knowledge  No knowledge  No knowledge  No knowledge  No knowledge  No knowledge  No knowledge | No knowledge  No knowledge  No knowledge  No knowledge  No knowledge  No knowledge  No knowledge  No knowledge  No knowledge  No knowledge  No knowledge  No knowledge  No knowledge  No knowledge  No knowledge | No knowledge  No knowledge  No knowledge  No knowledge  No knowledge  No knowledge  No knowledge  No knowledge  No knowledge  No knowledge  No knowledge  No knowledge  No knowledge  No knowledge  No knowledge | No knowledge  No knowledge  No knowledge  No knowledge  No knowledge  No knowledge  No knowledge  No knowledge  No knowledge  No knowledge  No knowledge  No knowledge  No knowledge  No knowledge  No knowledge | Activation  Activation  No knowledge  Activation  No knowledge  No knowledge  Inhibition  Inhibition  No knowledge  No knowledge  No knowledge  Phosphorylation  No knowledge  No knowledge  No knowledge |
| **Claude-Instant** | No knowledge  No knowledge  No knowledge  No knowledge  No knowledge  No knowledge  No knowledge  No knowledge  No knowledge  No knowledge  No knowledge  No knowledge  No knowledge  No knowledge  No knowledge | No knowledge  No knowledge  No knowledge  No knowledge  No knowledge  No knowledge  No knowledge  No knowledge  No knowledge  No knowledge  No knowledge  No knowledge  No knowledge  No knowledge  No knowledge | No knowledge  No knowledge  No knowledge  No knowledge  No knowledge  No knowledge  No knowledge  No knowledge  No knowledge  No knowledge  No knowledge  No knowledge  No knowledge  No knowledge  No knowledge | No knowledge  No knowledge  No knowledge  No knowledge  No knowledge  No knowledge  No knowledge  No knowledge  No knowledge  No knowledge  No knowledge  No knowledge  No knowledge  No knowledge  No knowledge | Activation  Activation  Inhibition  Activation  No knowledge  No knowledge  Inhibition  Inhibition  No knowledge  No knowledge  No knowledge  No knowledge  Inhibition  No knowledge  Phosphorylation |
| **Palm2** | No knowledge  No knowledge  No knowledge  No knowledge  No knowledge  No knowledge  No knowledge  No knowledge  No knowledge  No knowledge  No knowledge  No knowledge  No knowledge  No knowledge  No knowledge | No knowledge  No knowledge  No knowledge  No knowledge  No knowledge  No knowledge  No knowledge  No knowledge  No knowledge  No knowledge  No knowledge  No knowledge  No knowledge  No knowledge  No knowledge | No knowledge  No knowledge  No knowledge  No knowledge  No knowledge  No knowledge  No knowledge  No knowledge  No knowledge  No knowledge  No knowledge  No knowledge  No knowledge  No knowledge  No knowledge | No knowledge  No knowledge  No knowledge  No knowledge  No knowledge  No knowledge  No knowledge  No knowledge  No knowledge  No knowledge  No knowledge  No knowledge  No knowledge  No knowledge  No knowledge | Activation  Inhibition  Activation  No knowledge  Inhibition  No knowledge  Inhibition  Activation  Inhibition  No knowledge  No knowledge  No knowledge  No knowledge  Phosphorylation  Phosphorylation |
| **Bard** | No knowledge  No knowledge  No knowledge  No knowledge  No knowledge  No knowledge  No knowledge  No knowledge  No knowledge  No knowledge  No knowledge  No knowledge  No knowledge  No knowledge  No knowledge | No knowledge  No knowledge  No knowledge  No knowledge  No knowledge  No knowledge  No knowledge  No knowledge  No knowledge  No knowledge  No knowledge  No knowledge  No knowledge  No knowledge  No knowledge | No knowledge  No knowledge  No knowledge  No knowledge  No knowledge  No knowledge  No knowledge  No knowledge  No knowledge  No knowledge  No knowledge  No knowledge  No knowledge  No knowledge  No knowledge | No knowledge  No knowledge  No knowledge  No knowledge  No knowledge  No knowledge  No knowledge  No knowledge  No knowledge  No knowledge  No knowledge  No knowledge  No knowledge  No knowledge  No knowledge | Activation  Activation  Activation  No knowledge  No knowledge  No knowledge  Inhibition  Inhibition  No knowledge  No knowledge  No knowledge  No knowledge  Phosphorylation  Phosphorylation  Phosphorylation |
| **Codellama-34** | No knowledge  No knowledge  No knowledge  No knowledge  No knowledge  No knowledge  No knowledge  No knowledge  No knowledge  No knowledge  No knowledge  No knowledge  No knowledge  No knowledge  No knowledge | No knowledge  No knowledge  No knowledge  No knowledge  No knowledge  No knowledge  No knowledge  No knowledge  No knowledge  No knowledge  No knowledge  No knowledge  No knowledge  No knowledge  No knowledge | No knowledge  No knowledge  No knowledge  No knowledge  No knowledge  No knowledge  No knowledge  No knowledge  No knowledge  No knowledge  No knowledge  No knowledge  No knowledge  No knowledge  No knowledge | Activation  No knowledge  Inhibition  No knowledge  No knowledge  No knowledge  Inhibition  Activation  No knowledge  No knowledge  No knowledge  No knowledge  Activation  Phosphorylation  Phosphorylation | No knowledge  No knowledge  Activation  Activation  No knowledge  Inhibition  No knowledge  Inhibition  Activation  No knowledge  No knowledge  Activation  No knowledge  Phosphorylation  Phosphorylation |
| **Wizardlm-70** | No knowledge  No knowledge  No knowledge  No knowledge  No knowledge  No knowledge  No knowledge  No knowledge  No knowledge  No knowledge  No knowledge  No knowledge  No knowledge  No knowledge  No knowledge | No knowledge  No knowledge  No knowledge  No knowledge  No knowledge  No knowledge  No knowledge  No knowledge  No knowledge  No knowledge  No knowledge  No knowledge  No knowledge  No knowledge  No knowledge | No knowledge  No knowledge  No knowledge  No knowledge  No knowledge  No knowledge  No knowledge  No knowledge  No knowledge  No knowledge  No knowledge  No knowledge  No knowledge  No knowledge  No knowledge | Activation  No knowledge  No knowledge  Inhibition  No knowledge  No knowledge  Inhibition  No knowledge  No knowledge  Activation  No knowledge  Inhibition  No knowledge  No knowledge  Phosphorylation | Inhibition  Activation  Activation  Inhibition  No knowledge  No knowledge  Inhibition  Inhibition  No knowledge  No knowledge  Inhibition  No knowledge  Activation  Phosphorylation  Phosphorylation |
| **Wizardlm-13** | No knowledge  No knowledge  No knowledge  No knowledge  No knowledge  No knowledge  No knowledge  No knowledge  No knowledge  No knowledge  No knowledge  No knowledge  No knowledge  No knowledge  No knowledge | No knowledge  No knowledge  No knowledge  No knowledge  No knowledge  No knowledge  No knowledge  No knowledge  No knowledge  No knowledge  No knowledge  No knowledge  No knowledge  No knowledge  No knowledge | No knowledge  No knowledge  No knowledge  No knowledge  No knowledge  No knowledge  No knowledge  No knowledge  No knowledge  No knowledge  No knowledge  No knowledge  No knowledge  No knowledge  No knowledge | Activation  Activation  No knowledge  Inhibition  Inhibition  No knowledge  Inhibition  Inhibition  No knowledge  Activation  No knowledge  No knowledge  No knowledge  No knowledge  No knowledge | Activation  Inhibition  Activation  Activation  No knowledge  No knowledge  Inhibition  Inhibition  No knowledge  Activation  Inhibition  No knowledge  Activation  No knowledge  No knowledge |
| **Falcon-180** | No knowledge  No knowledge  No knowledge  No knowledge  No knowledge  No knowledge  No knowledge  No knowledge  No knowledge  No knowledge  No knowledge  No knowledge  No knowledge  No knowledge  No knowledge | No knowledge  No knowledge  No knowledge  No knowledge  No knowledge  No knowledge  No knowledge  No knowledge  No knowledge  No knowledge  No knowledge  No knowledge  No knowledge  No knowledge  No knowledge | No knowledge  No knowledge  No knowledge  No knowledge  No knowledge  No knowledge  No knowledge  No knowledge  No knowledge  No knowledge  No knowledge  No knowledge  No knowledge  No knowledge  No knowledge | Activation  Activation  No knowledge  Inhibition  No knowledge  No knowledge  Inhibition  Inhibition  No knowledge  No knowledge  Inhibition  Activation  No knowledge  No knowledge  Phosphorylation | Activation  Activation  Inhibition  Activation  Inhibition  No knowledge  Inhibition  Inhibition  Activation  No knowledge  Activation  No knowledge  Phosphorylation  No knowledge  Phosphorylation |
| **Mistral-7** | No knowledge  No knowledge  No knowledge  No knowledge  No knowledge  No knowledge  No knowledge  No knowledge  No knowledge  No knowledge  No knowledge  No knowledge  No knowledge  No knowledge  No knowledge | No knowledge  No knowledge  No knowledge  No knowledge  No knowledge  No knowledge  No knowledge  No knowledge  No knowledge  No knowledge  No knowledge  No knowledge  No knowledge  No knowledge  No knowledge | No knowledge  No knowledge  No knowledge  No knowledge  No knowledge  No knowledge  No knowledge  No knowledge  No knowledge  No knowledge  No knowledge  No knowledge  No knowledge  No knowledge  No knowledge | Activation  Activation  No knowledge  No knowledge  No knowledge  No knowledge  Activation  Activation  No knowledge  No knowledge  Activation  Activation  No knowledge  No knowledge  No knowledge | Activation  Inhibition  Activation  No knowledge  Inhibition  No knowledge  No knowledge  Inhibition  Inhibition  No knowledge  No knowledge  Activation  Inhibition  No knowledge  No knowledge |
| **Chatglm2-6** | No knowledge  No knowledge  No knowledge  No knowledge  No knowledge  No knowledge  No knowledge  No knowledge  No knowledge  No knowledge  No knowledge  No knowledge  No knowledge  No knowledge  No knowledge | No knowledge  No knowledge  No knowledge  No knowledge  No knowledge  No knowledge  No knowledge  No knowledge  No knowledge  No knowledge  No knowledge  No knowledge  No knowledge  No knowledge  No knowledge | No knowledge  No knowledge  No knowledge  No knowledge  No knowledge  No knowledge  No knowledge  No knowledge  No knowledge  No knowledge  No knowledge  No knowledge  No knowledge  No knowledge  No knowledge | Activation  Activation  No knowledge  Inhibition  No knowledge  No knowledge  Inhibition  No knowledge  No knowledge  No knowledge  No knowledge  Inhibition  No knowledge  No knowledge  Phosphorylation | Activation  Activation  No knowledge  Inhibition  Inhibition  No knowledge  No knowledge  Inhibition  Inhibition  No knowledge  No knowledge  No knowledge  Inhibition  No knowledge  Phosphorylation |
| **Vicuna-7b** | No knowledge  No knowledge  No knowledge  No knowledge  No knowledge  No knowledge  No knowledge  No knowledge  No knowledge  No knowledge  No knowledge  No knowledge  No knowledge  No knowledge  No knowledge | No knowledge  No knowledge  No knowledge  No knowledge  No knowledge  No knowledge  No knowledge  No knowledge  No knowledge  No knowledge  No knowledge  No knowledge  No knowledge  No knowledge  No knowledge | No knowledge  No knowledge  No knowledge  No knowledge  No knowledge  No knowledge  No knowledge  No knowledge  No knowledge  No knowledge  No knowledge  No knowledge  No knowledge  No knowledge  No knowledge | Activation  Activation  No knowledge  No knowledge  No knowledge  No knowledge  Inhibition  Inhibition  No knowledge  No knowledge  No knowledge  No knowledge  No knowledge  Phosphorylation Phosphorylation | Activation  Activation  Inhibition  No knowledge  No knowledge  Inhibition  Inhibition  No knowledge  Activation  No knowledge  Phosphorylation  No knowledge  Activation  Activation  No knowledge |
| **Vicuna-33** | No knowledge  No knowledge  No knowledge  No knowledge  No knowledge  No knowledge  No knowledge  No knowledge  No knowledge  No knowledge  No knowledge  No knowledge  No knowledge  No knowledge  No knowledge | No knowledge  No knowledge  No knowledge  No knowledge  No knowledge  No knowledge  No knowledge  No knowledge  No knowledge  No knowledge  No knowledge  No knowledge  No knowledge  No knowledge  No knowledge | No knowledge  No knowledge  No knowledge  No knowledge  No knowledge  No knowledge  No knowledge  No knowledge  No knowledge  No knowledge  No knowledge  No knowledge  No knowledge  No knowledge  No knowledge | Activation  No knowledge  Activation  No knowledge  No knowledge  No knowledge  No knowledge  Inhibition  Inhibition  No knowledge  Activation  No knowledge  No knowledge  No knowledge  Phosphorylation | Activation  Activation  No knowledge  Inhibition  No knowledge  Inhibition  Inhibition  Activation  No knowledge  No knowledge  Phosphorylation  No knowledge  No knowledge  No knowledge  No knowledge |
| **Vicuna-13b** | No knowledge  No knowledge  No knowledge  No knowledge  No knowledge  No knowledge  No knowledge  No knowledge  No knowledge  No knowledge  No knowledge  No knowledge  No knowledge  No knowledge  No knowledge | No knowledge  No knowledge  No knowledge  No knowledge  No knowledge  No knowledge  No knowledge  No knowledge  No knowledge  No knowledge  No knowledge  No knowledge  No knowledge  No knowledge  No knowledge | No knowledge  No knowledge  No knowledge  No knowledge  No knowledge  No knowledge  No knowledge  No knowledge  No knowledge  No knowledge  No knowledge  No knowledge  No knowledge  No knowledge  No knowledge | No knowledge  No knowledge  Activation  Activation  No knowledge  No knowledge  No knowledge  Inhibition  Inhibition  No knowledge  No knowledge  Inhibition No knowledge  No knowledge  Phosphorylation | Activation  Activation  No knowledge  Inhibition  No knowledge  Inhibition  Inhibition  No knowledge  No knowledge  No knowledge  Phosphorylation  Phosphorylation  No knowledge  No knowledge  No knowledge |
| **llama2-70b** | No knowledge  No knowledge  No knowledge  No knowledge  No knowledge  No knowledge  No knowledge  No knowledge  No knowledge  No knowledge  No knowledge  No knowledge  No knowledge  No knowledge  No knowledge | No knowledge  No knowledge  No knowledge  No knowledge  No knowledge  No knowledge  No knowledge  No knowledge  No knowledge  No knowledge  No knowledge  No knowledge  No knowledge  No knowledge  No knowledge | No knowledge  No knowledge  No knowledge  No knowledge  No knowledge  No knowledge  No knowledge  No knowledge  No knowledge  No knowledge  No knowledge  No knowledge  No knowledge  No knowledge  No knowledge | Activation  Activation  No knowledge  No knowledge  Inhibition  No knowledge  Inhibition  Inhibition  No knowledge  No knowledge  No knowledge  No knowledge  Inhibition  No knowledge  No knowledge | Activation  Activation  Inhibition  Inhibition  No knowledge  No knowledge  Inhibition  Inhibition  No knowledge  Activation  Activation  No knowledge  Phosphorylation  No knowledge  Phosphorylation |
| **llama2-13b** | No knowledge  No knowledge  No knowledge  No knowledge  No knowledge  No knowledge  No knowledge  No knowledge  No knowledge  No knowledge  No knowledge  No knowledge  No knowledge  No knowledge  No knowledge | No knowledge  No knowledge  No knowledge  No knowledge  No knowledge  No knowledge  No knowledge  No knowledge  No knowledge  No knowledge  No knowledge  No knowledge  No knowledge  No knowledge  No knowledge | No knowledge  No knowledge  No knowledge  No knowledge  No knowledge  No knowledge  No knowledge  No knowledge  No knowledge  No knowledge  No knowledge  No knowledge  No knowledge  No knowledge  No knowledge | Inhibition  Inhibition  Activation  Activation  No knowledge  No knowledge  No knowledge  Inhibition  Inhibition  No knowledge  Phosphorylation  No knowledge  No knowledge  No knowledge  No knowledge | Activation  Activation  No knowledge  No knowledge  No knowledge  Inhibition  Inhibition  No knowledge  Activation  No knowledge  Phosphorylation  Phosphorylation  No knowledge  No knowledge  No knowledge |
| **llama2-7b** | No knowledge  No knowledge  No knowledge  No knowledge  No knowledge  No knowledge  No knowledge  No knowledge  No knowledge  No knowledge  No knowledge  No knowledge  No knowledge  No knowledge  No knowledge | No knowledge  No knowledge  No knowledge  No knowledge  No knowledge  No knowledge  No knowledge  No knowledge  No knowledge  No knowledge  No knowledge  No knowledge  No knowledge  No knowledge  No knowledge | No knowledge  No knowledge  No knowledge  No knowledge  No knowledge  No knowledge  No knowledge  No knowledge  No knowledge  No knowledge  No knowledge  No knowledge  No knowledge  No knowledge  No knowledge | Activation  Activation  No knowledge  Inhibition  No knowledge  No knowledge  Inhibition  Inhibition  No knowledge  No knowledge  No knowledge  No knowledge  No knowledge  Phosphorylation  No knowledge | Activation  Activation  Activation  No knowledge  No knowledge  No knowledge  Inhibition  Inhibition  No knowledge  No knowledge  Inhibition  Phosphorylation  Phosphorylation  No knowledge  No knowledge |
| **Qwen-14b** | No knowledge  No knowledge  No knowledge  No knowledge  No knowledge  No knowledge  No knowledge  No knowledge  No knowledge  No knowledge  No knowledge  No knowledge  No knowledge  No knowledge  No knowledge | No knowledge  No knowledge  No knowledge  No knowledge  No knowledge  No knowledge  No knowledge  No knowledge  No knowledge  No knowledge  No knowledge  No knowledge  No knowledge  No knowledge  No knowledge | No knowledge  No knowledge  No knowledge  No knowledge  No knowledge  No knowledge  No knowledge  No knowledge  No knowledge  No knowledge  No knowledge  No knowledge  No knowledge  No knowledge  No knowledge | Activation  Activation  No knowledge  No knowledge  No knowledge  No knowledge  Activation  Activation  No knowledge  No knowledge  No knowledge  No knowledge  No knowledge  No knowledge  No knowledge | Activation  Activation  No knowledge  Inhibition  No knowledge  No knowledge  Inhibition  Inhibition  No knowledge  No knowledge  Activation  No knowledge  Inhibition  No knowledge  No knowledge |

**Table S6:** Structured Prompts for determining using KEGG Pathway

| **Prompt1** | **Prompt3** |
| --- | --- |
| As a gene interaction analyst, your task is to determine the effect of {gene1} on {gene2} within the context of the KEGG Pathway Database, which represents our knowledge of molecular interaction, reaction, and relation networks for various biological processes. Provide a concise and definitive answer using one of the following terms: activation (gene1 activates gene2), inhibition (gene1 inhibits gene2), phosphorylation (gene1 phosphorylates gene2), or no information (no known relationship). Make sure your answer is definitive, composed of 'activation', 'inhibition', 'phosphorylation' or 'no information without further details or explanation. | As a gene interaction specialist, your task is to determine the effect of {gene1} on {gene2} using the KEGG Pathway Database, a comprehensive resource for understanding molecular interaction and relation networks in cellular processes. Provide a clear and unambiguous answer using one of the following terms: 'activation' (gene1 activates gene2), 'inhibition' (gene1 inhibits gene2), 'phosphorylation' (gene1 phosphorylates gene2), or 'no information' if there is no known relationship. Ensure your answer is definitive and based on the current knowledge in the KEGG Pathway Database, composed of 'activation', 'inhibition’,’ phosphorylation', or 'no information' without further details or explanation. |
| **Prompt3** | **Prompt4** |
| As a gene expression analyst, you are tasked with determining the effect of {gene1} on {gene2} using the KEGG Pathway Database, a valuable resource for understanding molecular interaction networks in biological processes. Your answer should only include one of the following terms: 'activation' (gene1 activates gene2), 'inhibition' (gene1 inhibits gene2), or 'phosphorylation' (gene1 phosphorylates gene2). Make sure your answer is definitive, composed of 'activation', 'inhibition', 'phosphorylation' or 'no information without further details or explanation. | As a genomics analyst, your task is to determine the effect of {gene1} on {gene2} using the KEGG Pathway Database, a comprehensive resource for understanding molecular interaction networks involved in cellular processes. Provide a clear and unambiguous answer using one of the following terms: 'activation' (gene1 activates gene2), 'inhibition' (gene1 inhibits gene2), or 'phosphorylation' (gene1 phosphorylates gene2). Ensure your answer is definitive and composed of 'activation', 'inhibition', 'phosphorylation', or 'no information without further details or explanation. |
| **Prompt5** | **Prompt6** |
| As a network biologist, your task is to determine the effect of {gene1} on {gene2} using the KEGG Pathway Database, a comprehensive resource for understanding molecular interaction networks in various biological processes. Provide a clear and unambiguous answer using one of the following terms: 'activation' (gene1 activates gene2), 'inhibition' (gene1 inhibits gene2), or 'phosphorylation' (gene1 phosphorylates gene2). Ensure your answer is based on the current knowledge in the KEGG Pathway Database and composed of 'activation', 'inhibition', or 'phosphorylation' without further details or explanation. Make sure your answer is definitive, composed of 'activation', 'inhibition', 'phosphorylation' or 'no information without further details or explanation. | As a system biologist, your task is to determine the effect of {gene1} on {gene2} using the KEGG Pathway Database, a comprehensive resource for understanding molecular interaction and relation networks in cellular processes. Provide a clear and unambiguous answer using one of the following terms: 'activation' (gene1 activates gene2), 'inhibition' (gene1 inhibits gene2), 'phosphorylation' (gene1 phosphorylates gene2), or 'no information' if there is no known relationship. Ensure your answer is definitive and composed of 'activation', 'inhibition', 'phosphorylation', or 'no information without further details or explanation. |
| **Prompt7 = Final prompt**  As a computational biologist, determine what is the relation between {gene1} and {gene2} in KEGG Pathway Database. KEGG pathways are collections of pathway maps that represent our knowledge of the molecular interaction, reaction, and relation networks for various biological processes. To determine the relation between {gene1} and {gene2}, please provide a definitive answer without further thought. Describe the relation in one or two of the following words: activation, inhibition, phosphorylation. If you are replying with two relations, separate two relations using '...'. Please note that the directionality of the relationship is important. Make sure your answer is definitive, answer with relations only, without further details or explanations. Make sure your answer is definitive, composed of 'activation', 'inhibition', 'phosphorylation' or 'no information without further details or explanation. | |

**Table S7:** Matched genes between LLM predictions and the ground-truth genes in Adherens junction

| **Model Prediction** | **Ground -Truth** | | | | | | | | | | | | | | |
| --- | --- | --- | --- | --- | --- | --- | --- | --- | --- | --- | --- | --- | --- | --- | --- |
|  | CDH1 | CTNNB1 | CTNNA | CTNND1 | CDC42 | CDH3 | ACTN4 | ACTB | EP300 | FGFR1 | PARD3 | MET | LEF1 | SMAD4 | Average Ratio |
| **Claude** | 0.5 | 0.3 | 0.3 | 0 | 0 | 0.4 | 0 | 0 | 0 | 0 | 0 | 0 | 0 | 0 | 0.1071 |
| **Claude Instant** | 0.5 | 0.4 | 0.2 | 0.1 | 0 | 0.5 | 0 | 0 | 0 | 0 | 0 | 0 | 0 | 0 | 0.1214 |
| **Cohere Weight** | 0.4 | 0.2 | 0 | 0.2 | 0 | 0.4 | 0.3 | 0 | 0 | 0 | 0.3 | 0 | 0 | 0 | 0.1286 |
| **GPT-3.5** | 0.5 | 0.3 | 0 | 0.2 | 0.2 | 0 | 0 | 0 | 0 | 0 | 0 | 0 | 0 | 0 | 0.0857 |
| **GPT-4** | 0.5 | 0.4 | 0 | 0.4 | 0.3 | 0.5 | 0 | 0.3 | 0 | 0.2 | 0.2 | 0 | 0 | 0 | 0.2000 |
| **Claude2-Pro** | 0.5 | 0.4 | 0 | 0.4 | 0.3 | 0.5 | 0 | 0.3 | 0.2 | 0.2 | 0.2 | 0 | 0 | 0 | 0.2143 |
| **Palm-2** | 0.3 | 0 | 0 | 0.2 | 0 | 0.3 | 0 | 0.1 | 0 | 0 | 0 | 0 | 0 | 0 | 0.0643 |
| **Bard** | 0.5 | 0.4 | 0 | 0.3 | 0 | 0.5 | 0 | 0.3 | 0 | 0 | 0.2 | 0 | 0 | 0 | 0.1571 |
| **Codellama-34 *** | 0.3 | 0.2 | 0 | 0.2 | 0 | 0.3 | 0 | 0 | 0 | 0 | 0 | 0 | 0 | 0 | 0.0714 |
| **Wizardlm-70b *** | 0.3 | 0.3 | 0 | 0 | 0 | 0 | 0 | 0 | 0 | 0 | 0 | 0 | 0 | 0 | 0.0429 |
| **Wizardlm-13b *** | 0.3 | 0.3 | 0.2 | 0 | 0 | 0.3 | 0 | 0 | 0 | 0 | 0 | 0 | 0 | 0 | 0.0786 |
| **Falcon-180b *** | 0.3 | 0.2 | 0 | 0.2 | 0.2 | 0.3 | 0 | 0 | 0 | 0 | 0 | 0 | 0 | 0 | 0.0857 |
| **Mistral-7b *** | 0.2 | 0.2 | 0 | 0 | 0 | 0.2 | 0 | 0.1 | 0 | 0 | 0 | 0 | 0 | 0 | 0.0500 |
| **Chatglm2-6b*** | 0.2 | 0.1 | 0 | 0 | 0 | 0 | 0 | 0 | 0 | 0 | 0 | 0 | 0 | 0 | 0.0214 |
| **Vicuna-7b *** | 0.4 | 0.3 | 0.2 | 0.3 | 0 | 0.4 | 0 | 0 | 0 | 0 | 0 | 0 | 0 | 0 | 0.1143 |
| **Vicuna-33b *** | 0.3 | 0.2 | 0.2 | 0.1 | 0 | 0.3 | 0 | 0 | 0 | 0 | 0 | 0 | 0 | 0 | 0.0786 |
| **Vicuna-13b *** | 0.2 | 0.1 | 0.1 | 0 | 0 | 0 | 0 | 0.1 | 0 | 0 | 0 | 0 | 0 | 0 | 0.0357 |
| **llama2-70b *** | 0.4 | 0 | 0.3 | 0 | 0 | 0 | 0 | 0 | 0 | 0 | 0 | 0 | 0 | 0 | 0.0500 |
| **llama2-13b *** | 0.3 | 0.2 | 0.1 | 0 | 0 | 0.3 | 0 | 0 | 0 | 0 | 0 | 0 | 0 | 0 | 0.0643 |
| **llama2-7b *** | 0.2 | 0.1 | 0.1 | 0.1 | 0 | 0 | 0 | 0 | 0 | 0 | 0 | 0 | 0 | 0 | 0.0357 |
| **Qwen-14b *** | 0.2 | 0.2 | 0 | 0 | 0 | 0 | 0 | 0 | 0 | 0 | 0 | 0 | 0 | 0 | 0.0286 |
| **Gene Ratio (API Models)** | 0.463 | 0.300 | 0.063 | 0.225 | 0.100 | 0.388 | 0.038 | 0.125 | 0.025 | 0.050 | 0.113 | 0.00 | 0.000 | 0.000 | 0.135 |
| **Gene Ratio (Open-source Models** | 0.277 | 0.185 | 0.092 | 0.069 | 0.015 | 0.162 | 0.000 | 0.015 | 0.000 | 0.000 | 0.000 | 0.00 | 0.000 | 0.000 | 0.0582 |
| **Gene Ratio (All models)** | 0.348 | 0.229 | 0.081 | 0.129 | 0.048 | 0.248 | 0.014 | 0.057 | 0.010 | 0.019 | 0.043 | 0.00 | 0.00 | 0.000 | 0.0875 |

The number for each gene by each LLM represents the ratio of correct recognitions in 10 runs. The bottom three rows show the average ratio of the gene among API models, open-source models (marked by *), and all models. The right-most column indicates the average ratio of each LLM.

**Table S8**: Predicted genes not present in Tight junction by the 21 LLMs.

| **Model** | **# of Unmatched Genes** | **Unmatched Genes** |
| --- | --- | --- |
| Claude | 11 | OCL, TJP1, ACTB, CTNNB1, JA1, CTN, MO9, ACT, PTPN1, PTPN2, PRKCA |
| Claude-Instant | 11 | TJP1, 7H6, JA-1, MYH9, RHOA, RAG1, CRB2, CAV1, TJP2, MAGI1, TJP2 |
| Cohere | 9 | PARP2, POD, YAM-1, CAV1, KAI1, CLRN3, GZMB, F1R, TTLL7 |
| GPT-3.5 | 11 | Zo, TJP1, TJP2, MAGI1, RJM-1, CGAL1, MLLT4, MPP5, CASK, PP5 ,PARD |
| GPT-4 | 9 | MPP5, CASK, HAM-1, CTNNA1, MPP5, PARD6G, TJP1, MAGI1, ZO-2 |
| Claude-Pro | 9 | TJP1, Zo1, HAM7, NNA1, PP5, RD6G, JP1, TJP1, ZO-2 |
| Palm-2 | 13 | ACTB, AFANP, BCL1, CKND5, JAT1, AMB, ADG, ACTA2, ACTA3, CLD20, BCL, ATP, CLND3 |
| Bard | 11 | DLG3, ZO-1, MYL12B, CLDN8, CKND, FAYR, BCL, PARD6G, TJP1, MAGI1, CLND |
| Codellama-34 | 11 | POD, CD2, MPP5, FAM-1, C22N, CAAG, RA3, TJP1, MAGI1, TJP1, CAV1 |
| Wizardlm-70 | 9 | MPP5, CASK, MPP5, M1A, CGAL1, MLLT4, MPP5, CASK, ARD6G |
| Wizardlm-13 | 13 | CLDN2, CLDN3, CLDN4, CLDN5, C4BC, JAM-1, A2MB, CTNNA1, MPP5, PARD6G, TJP1, MAGI1, TJP1 |
| Falcon-180b | 9 | TJP1, Zo1, CTNNB1, JAM-1, P2BC, TJP1, MAGI1, CLND, RA3 |
| Mistral-7b | 12 | CLDN4, CLDN4, ACTB, B2MQ, AMB, LA4C, CRB2, CAV1, TJP2, MAGI1, TJP2, CRB2 |
| Chatglm2-6 | 11 | MPP5, P1P5, JA1, NNA1, PFP5, NNA1, H2SK, NNA , S2KDD1, CTN, CASK |
| Vicuna-7b | 10 | ACTB, MPP5, AMB, ADG, RD6G, JP1, TJP1, ADG, MAGI1, TJUP2 |
| Vicuna-33b | 10 | BCN1, PARD6G , RA3, H2PE, ZNA1, MLLT4, NNA ,LA4C , T2TN, FAGI1 |
| Vicuna-13b | 10 | MPP5, ACTB, NNA1, PP5, NNA1, ASTK2, NNA , NNA1, CRTN, CASK |
| llama2-70b | 11 | CDC2, CD2, MPP5, P2AR, AM1, MPP5, PARD6G, TJP1, MAGI1, TJP1, TJP2 |
| llama2-13b | 10 | Zo1, CTNNB1, PP5, PP5, NNA1, CASK, NNA , P2AR , CTN, , CTNNA1 |
| llama2-7b | 10 | CD2, MPP5, PP5, NNA1, C22N , X2GA, CASK, MP2AP , ,TNNA1, R2SK |
| Qwen-14b | 12 | CDC2, CD1, TJP2, MAGI1, JA, CGAL1, MLLT4, MPP5, CASK, CTN, NNA , TJP2 |

**Table S9**: Predicted genes not present in the gap junction by the 21 LLMs.

| **Model** | **# of Unmatched Genes** | **Unmatched Genes** |
| --- | --- | --- |
| Claude | 11 | GJD1, CX33, CX36, CX45, GJA4, CX59, CX62, GJA8, CX40, CX47, CX4 |
| Claude-Instant | 11 | GJC3, SLC24A3, SLC24A6, PRPH2, LIM2, CX62, GJB6, SLC2, GNJB, C24A3, PH2 |
| Cohere | 10 | GJC3, SLC24A6, ITGB1, GJA8, CL3, CLCN3, CLCN1, GJB6, C24A3, GJA10 |
| GPT-3.5 | 11 | GJA12, GJB10, GJB7, GJA7, GJB, C24A3, GSJB3, GJAC, GJA8, G2DD, GJAA |
| GPT-4 | 9 | GBJB6, GYJB8, GJTB3, GJA4, GJB, GJDD, GJB, CLCN, G2JA |
| Claude-Pro | 9 | GJB6, GJB8, GJB3, GJA4, GJB, GJDD, GJB, PRKG1, GBJA |
| Palm2 | 12 | GJ2, CX33, PRPH2, LIM, CLP, COROM1, CLSC3, BCAP3, CA, COPORS, COROS, RODS |
| Bard | 10 | NXI22, PANX2, PAN2X3, GJML, GJB, C24A6, ITGB1, GJA8, CLCN, GJA |
| Codellama-34 | 10 | GJC3, SLC24A3, COROM1, PRPH2, CDSM2, CX62, GJB6, SLC2, SLC2, GJB |
| Wizardlm-70 | 9 | SLC24A3, CX62, PRPH2, LIM2, CX62, GJB6, SLC2, SLC2, GRJB |
| Wizardlm-13 | 11 | GJBC ,GJB7, GJA7, GJB, GJB2, GJB3, GJAC, GJB7, PRP, GJB7, GJA7 |
| Falcon-180b | 10 | GJC3, SL24A3, CDSM2, PRPH2, LIM2, CX62, GJB6, SLC2, CLCN, GXJA |
| Mistral-7b | 11 | GJC3, RPH2, C24A6, PRPH2, LIM2, CX62, GJB6, GFJC3, SLC3, GJB7, GJA7 |
| Chatglm2-6 | 12 | GJB10, GJB7, GJA7, GEJB, GVJB2, GTJB3, GCJAC, GJA8, GJB10, GJB7, GJA7, GJ4B |
| Vicuna-7b | 9 | C24A3, DSM2, PRPH2, LIM2, CX62, GJB6, SLC2, GJA7, GAEJB |
| Vicuna-33b | 8 | RLC2A, CX62, PRPH2, SLC24A6, ITGB1, GJA8, CLCN, SJA |
| Vicuna-13b | 10 | GJA7, GTJB, GTJB2, GJYB3, GJAC, GJA8, GJB10, GJB7, GJA7, GEJB |
| llama2-70b | 11 | GJB7, GJA7, PRPH2, GJB2, GJB3, FJAC, GJB7, PPH2, GJB7, CLCN, SJA |
| llama2-13b | 9 | ITGB1, GJB, GJB2, DYB3, GJAC, GJA8, CX62, GJA7, GJB |
| llama2-7b | 8 | RPH2, GJB2, GJB3, GJAC, GJA8, GJB10, CLCN, CGJA |
| Qwen-14b | 14 | GJB, GJC, GJB10, G7, CLCN, GJB, GJ2, RPH2, GJAC, GJA8, GA5, D22D, GJAA SLC2 |

**Table S10**: Predicted genes not present in cellular senescence by the 21 LLMs.

| **Model** | **# of Unmatched Genes** | **Unmatched Genes** |
| --- | --- | --- |
| Claude | 12 | CDKN1B, DEC1, TP53, ETS2, ETS1/2, IL6, DEC1, PAI-1, DEC1, LMNB1, SO, CCNA2 |
| Claude-Instant | 12 | TP53, TGFB1, RELA, IGF1R, IL1A, IKBKB, FOXO1, CREB1, MDM2, PAI1, IKBKB, MDM2 |
| Cohere | 10 | CDK4, COX4II, MMP11, FOXO4, RBL2, TIMP, MYC, TUG, SKP2, RPL10A |
| GPT-3.5 | 12 | BKT, CHEK2, HMGA1, RB1, FOXO, NRF2, BCL2, AKT1, HIF1A, PTEN, IL-6, NFKB |
| GPT-4 | 9 | CDK4, mTOR, NF-dB, NRF2, p38, CCNA2, IL-6, SIRT7, CCNE2 |
| Claude-Pro | 9 | TERT, CDC2, NF, IL6/IL8, RB1, ETSI, TP53, PTEN, PML |
| Palm2 | 11 | P53, P16INK, SODI, BCL, FOXO3A, NRF2, Mkk9, AKT2, DDB2, E2F6, FAS |
| Bard | 9 | HAM, mTOR, NF-dB, P16INK, RB1, ETSI, TP53, PTEN, PML |
| Codellama-34 | 11 | TERT, CDC2, NF, IL6/IL8, p38, CCNA2, IL-6, SIRT7, CCNE2, p8, CCNA2 |
| Wizardlm-70 | 12 | CDK, BKB, FOXO3A, NRF2, Mkk9, FOXO3A, RB1, ETSI, TP53, PTEN, PML, RB1 |
| Wizardlm-13 | 11 | CDK4, mTOR, NF-dB, NRF2, CDK4, mTOR, TIMP, MYC, TUG, SKP2, RPL10A |
| Falcon-180b | 11 | CDKN1B, CDK4, COX4II, MMP11, CDK4, BCL, FOXO3A, NRF2, Mkk9, AKT2, DDB2 |
| Mistral_7b | 11 | CDKN1B, CDKN1B, mTOR, NF-dB, P16INK, RB1, ETSI, TP53, PTEN, PML, mTOR |
| Chatglm2-6 | 14 | CHEK2, HMGA1, RB1, FOXO, NRF2, BCL2, AKT1, HIF1A, PTEN, IL-6, NFKB, CHEK2, HMGA1, RB1 |
| Vicuna-7b | 11 | CDKNA, COX4II, MMP11, CDK4, MP11, BCL, FOXO3A, NRF2, Mkk9, BCL, BCL |
| Vicuna-33b | 10 | CDKNA, DAM, NF-κB, P16INK, RB1, ETSI, TP53, PTEN, PML, mTOR |
| Vicuna-13b | 11 | CDK4, mTOR, NF-KB, NRF2, CDK4, mTOR, TIMP, MYC, TUG, SKP2, RPL10A |
| llama2-70b | 11 | RELA, IGF1R, IL1A, IKBKB, FOXO1, CREB1, MDM2, PAI1, IKBKB, MDM2, RELA |
| llama2-13b | 11 | mTOR, NF-κB, NRF2, CDK4, mTOR, TIMP, MYC, TUG, SKP2, RPL10A, mTOR |
| llama2-7b | 11 | IGF1R, mTOR, NF-KB, NRF2, CDK4, mTOR, TIMP, MYC, TUG, SKP2, RPL10A |
| Qwen-14b | 14 | CHEK2, HMGA1, RB1, FOXO, NRF2, BCL2, AKT1, HIF1A, PTEN, IL-6, NFKB, CHEK2, HMGA1, RB1 |

**Table S11:** Predicted genes not present in phagosome by the 21 LLMs

| **Model** | **# of Unmatched Genes** | **Unmatched Genes** |
| --- | --- | --- |
| Claude | 10 | ATP6V1B2, HLA-DRA, TUBB, HYAL1, ATP6V0D1, PIK3C3, TUBB, AMP2, ETS1/2, P6V1B2 |
| Claude-Instant | 11 | CD14, RAB5C, LR4, ADRA, YAL1, TY6V0D1, RAK4, FKB2, GALS3, IRS1, ITGB2 |
| Cohere | 10 | ATP6AP1, THBS1, HS3ST3, MPP1, ELABELA, C17f99, AP3M1, RAB7, ADRA, COPA |
| GPT-3.5 | 12 | CD14, CD68, CD14, RAB11A, PLD, SRC, TLRs, PI3K, LAMP1, NADPH, ITGAM, TGAM |
| GPT-4 | 8 | ATP6V1H, PLCG2, MO, CTSD, VTI1B, CTS, CYBA, LAMP1 |
| Claude-Pro | 9 | CDC42, CAT, CYBB, NCF, FOXO4, IL6, VAM, GA, GAM |
| Palm2 | 12 | ATG, ATG9, ATG, ATG6A, ATG16R, YAL1, TLRs, PI3K, LAMP1, LAMP1, NADPH, ITGAM |
| Bard | 10 | CDC42, PLCG2, CTSD, ELABELA, C17f99, AP3M1, RAB7, ELABELA, IRS1, IRS |
| Codellama-34 | 12 | THBS1, HS3ST3, MPP1, ELABELA, CY2GA, AP3M1, RAB7, THBS1, HS3ST3, IRS1, ITGB2, IRS1 |
| Wizardlm-70 | 11 | P3M1, HS3ST3, MPP1, ELABELA, CY2GA, AP3M1, RAB7, ACT, ADRA, COPA, HS3ST3 |
| Wizardlm-13 | 10 | TLRs, HS3ST3, MPP1, HS3ST3, MPP1, VTI1B, CTS, VTI1B, IRS1, ITGB2 |
| Falcon-180b | 9 | ATP6AP1, THBS1, HS3ST3, TLRs, PI3K, LAMP1, LAMP1, NADPH, ITGAM |
| Mistral-7b | 10 | V1B2, PLCG2, MO, CTSD, YAL1, TLRs, PI3K, LAMP1, IRS1, ITGB2 |
| Chatglm2-6 | 12 | CD14, CD68, CD14, RAB11A, PLD, SRC, TLRs, PI3K, LAMP1, LAMP1, NADPH, ITGAM |
| Vicuna-7b | 11 | PV1B2, HS3ST3, MP, NF1, YAL1, TLRs, LAMP1, AMP1, ACB, NADPH, ITGAM |
| Vicuna-33b | 12 | ITGB2, RA1, CD4, CD68, RAB11A, PLD, SRC, CD14, IRS1, ITGB2, ITGP, MPP |
| Vicuna-13b | 10 | ITGB2, RA1, HS3ST3, NC, YAL1, TLRs, LAMP1, MAB, NADPH, ITGAM |
| llama2-70b | 10 | MPP, HS3ST3, MPP1, ELABELA, C17f99, AP3M1, TAB7, ACT, LAMP1, IRS |
| llama2-13b | 11 | HS3ST3, MPP1, ELABELA, HC1y33, AP3M1, EA1B7, ACT, CD68, CD14, IRS1, ITGB2 |
| llama2-7b | 9 | ITGB2, HS3ST3, YAL1, TLRs, LAMP1, AB, ATB, NADPH, ITGAM |
| Qwen-14b | 13 | CD14, RA1, CD68, CD14, RAB11A, PLD, SRC, TLRs, PI3K, LAMP1, LAMP1, NADPH, ITGAM |

**Table S12**: Predicted genes not present in proteoglycans in cancer by the 21 LLMs.

| **Model** | **# of Unmatched Genes** | **Unmatched Genes** |
| --- | --- | --- |
| Claude | 7 | DCN, CHSY1, GPC3, BCAN, YLT1, HAS3, TGB3 |
| Claude-Instant | 8 | CSPG4, SDC4, HST3, LT25D1, GPC, PRG4, YLT1, GPC3 |
| Cohere | 7 | PTX3, OLFM4, LRP1, VSIG4, ITGB5, PRG4, GFB1I1 |
| GPT-3.5 | 9 | EGFR, CDKN2A, PIK3CA, HIF1A, PAX3, VEGFA, WNT, TGFBR, NF1 |
| GPT-4 | 6 | AGRN, ITGA2, MMP9, HSPG2, GPC1, EXT2 |
| Claude-Pro | 6 | CHSY3, CHST11, NDST1, EXT1, SULF1, HSY3 |
| Palm2 | 9 | ADAMA, TGFB1, MMP2, TGF, WNTB, WNT3A, RAS, SPO1, CTGF |
| Bard | 7 | VCAM, NCAM, PLU, HYAL, HS33, GPC3, GPC1 |
| Codellama-34 | 7 | PIK3CA, HIF1A, PAX3, VEGFA, WNT, TGFBR, HST1 |
| Wizardlm-70 | 9 | P6V1B2, CAM, MMP9, HSPG2, GPC1, HSPG2, ITGB5, PRG4, GFB1I1 |
| Wizardlm-13 | 8 | PLU, HYAL, PLU, HYAL, HS33, GPC3, PLU, CAM |
| Falcon-180b | 7 | ADAMA, TGFB1, EXT1, SULF1, EXT1, SULF1, AMF |
| Mistral-7b | 7 | ADAMA, TGFB1, CD, EXT1, SULF1, YAL, ULF1 |
| chatglm2-6 | 10 | EGFR, CDKN2A, PIK3CA, HIF1A, CD4, PAX3, VEGFA, WNT, TGFBR, DKN2 |
| Vicuna-7b | 7 | ADAMA, TGFB1, SULF1, EXT1, SULF1, SPO1, CTGF |
| Vicuna-33b | 7 | ADAMA, GFB1, CD4, EXT1, SULF1, AK1, CTGF |
| Vicuna-13b | 10 | CDKN2A, PIK3CA, HIF1A, CD4, HYAL, HS33, GPC3, PLU, AT1, PC3 |
| llama2-70b | 9 | ADAMA, TGFB1, CDC2, LT25D1, GPC, PRG4, YLT1, AKT, SULF1 |
| llama2-13b | 8 | ADAMA, TGFB1, CDC2, SULF1, EXT1, SULF1, AK1, CTGF |
| llama2-7b | 7 | ADAMA, TGFB1, CD4, T25D, GPC, PRG4, SULF |
| Qwen-14b | 9 | D63, ADAMA, TGFB1, PAX3, VEGFA, WNT, TGFBR, NF1, PAX3 |

**Table S13:** Predicted genes not present in autoimmune thyroid disease by the 21 LLMs.

| **Model** | **# of Unmatched Genes** | **Unmatched Genes** |
| --- | --- | --- |
| Claude | 6 | VDR, IL21, IRF4, IL0, IFNG, FCGR3A |
| Claude- Instant | 8 | PRKCQ, H2B3, C40, CCR6, RKCQ, CD25, CD80, BACH2 |
| Cohere | 7 | OLFM4, IL21, C40, BACH2, STAT4, CTLA4, FASLG |
| GPT-3.5 | 8 | PADI4, T, BACH2, CD25, IL0, FCRL3, IKZF1, IL-23R |
| GPT-4 | 6 | CTLA4, VDR, IL0, VDR, ABCB1, IFNG |
| Claude-pro | 6 | TO, AF6, IL1B, STAT4, IL17F, FCGR3A |
| Palm2 | 8 | PADI4, IFNG, AIRE, HLA, CTLA4, XIAP, TYP, XIAP |
| Bard | 8 | FM4, CD0, ACH2, TAT25, IL1, AITD, CTLA4, TNF |
| Codellama-34 | 8 | CCR6, RKCQ, IRF1, H2B3, CTLA4, ISG15, CD80, BACH2 |
| Wizardlm-70 | 8 | PADI4, AIRE, HLA, CTLA4, XIAP, TYP, XIAP, AIRE |
| Wizardlm-13 | 8 | OLFM4, IL21, XYZ, AIRE, HLA, H2B3, CTLA4, ISG15 |
| Falcon-180b | 7 | IL2, AIRE, HLA, IL1, XIAP, AIRE, XIAP |
| Mistral-7b | 8 | PADI4, HLA, CTLA4, XIAP, IL0, STAT4, CTLA4, XIAP |
| Chatglm2-6 | 9 | PAD, T, CD4, BACH2, CD25, IL0, FCRL3, IKZF1, IL-23R |
| Vicuna-7b | 8 | CCR6, RKCQ, AIRE, HLA, CTLA4, TYP, XIAP, TYP |
| Vicuna-33b | 7 | PAD, CD4, BACH2, IL0, FCRL3, IKZF1, IL-23R |
| Vicuna-13b | 7 | PADI4, HLA, CTLA4, XIAP, XIAP, AIRE, XIAP |
| llama2-70b | 7 | OLFM4, IL21, CTLA4, XIAP, TYP, XIAP, TP |
| llama2-13b | 7 | PADI4, HLA, CTLA4, XIAP, XIAP, AIRE, XIAP |
| llama2-7b | 6 | PADI4, HLA, CTLA4, XIAP, AIRE, XIAP |
| Qwen-14b | 9 | PADI4, Tog, CD0, BACH2, CD25, FL3, IKZF1, IL-23R, PADI4 |

**Table S14**: Comparative performance of 21 computational models in predicting gene regulatory relations and recognizing KEGG pathways, categorized as API-based and open-source models.

| Type | Model | Gene Regulatory Relations (F1) | Gene Regulatory  Relations (Rank) | KEGG Pathway Recognition (Mean) | KEGG Pathway Recognition (Rank) |
| --- | --- | --- | --- | --- | --- |
| API base | Claude | 0.4215 | 3 | 0.1890 | 5 |
|  | Claude-Instant | 0.3263 | 5 | 0.1519 | 6 |
|  | GPT-3.5 | 0.1485 | 8 | 0.1358 | 7 |
|  | Cohere | 0.3742 | 4 | 0.2089 | 3 |
|  | **GPT-4** | **0.4448** | **1** | **0.2778** | **1** |
|  | Claude-Pro | 0.4386 | 2 | 0.2657 | 2 |
|  | Palm2 | 0.2614 | 7 | 0.1048 | 8 |
|  | Bard | 0.2785 | 6 | 0.1923 | 4 |
| Open source | Codellama-34 | 0.1435 | 6 | 0.1622 | 5 |
|  | Wizardlm-70b | 0.0449 | 13 | 0.1345 | 8 |
|  | Wizardlm-13b | 0.0962 | 11 | 0.1459 | 7 |
|  | **Falcon-180b** | **0.2787** | **1** | **0.2237** | **1** |
|  | Mistral-7b | 0.1226 | 8 | 0.1428 | 9 |
|  | Chatglm2-6b | 0.1821 | 3 | 0.0881 | 12 |
|  | Vicuna-7b | 0.1225 | 9 | 0.1744 | 4 |
|  | Vicuna-33b | 0.1405 | 7 | 0.1658 | 6 |
|  | Vicuna-13b | 0.1806 | 4 | 0.1540 | 10 |
|  | llama-2-70b | 0.1583 | 5 | 0.1540 | 11 |
|  | llama-2-13b | 0.0912 | 12 | 0.1712 | 3 |
|  | llama-2-7b | 0.1923 | 2 | 0.2207 | 2 |
|  | Qwen-14b | 0.1013 | 10 | 0.0842 | 13 |
